# The diagnostic accuracy of cardiac ultrasound for acute myocardial ischemia in the emergency department: a systematic review and meta-analysis

**DOI:** 10.1186/s13049-024-01192-3

**Published:** 2024-03-11

**Authors:** Virginia Zarama, María Camila Arango-Granados, Ramiro Manzano-Nunez, James P. Sheppard, Nia Roberts, Annette Plüddemann

**Affiliations:** 1 Facultad de Ciencias de la Salud, Universidad ICESI, Cali, Colombia; 2 Department of Emergency Medicine, Fundación Valle del Lili, Carrera 98 # 18-49, 760032 Cali, Colombia; 3 Nuffield Department of Primary Care Health Sciences and the Department for Continuing Education, University of Oxford, Oxford, Oxfordshire, UK; 4 Clinical Research Unit, Hospital del Mar Research Institute, Barcelona, Spain; 5 Centre for Evidence-Based Medicine, Nuffield Department of Primary Care Health Sciences, University of Oxford, Oxford, Oxfordshire, UK; 6 Bodleian Health Care Libraries, University of Oxford, Oxfordshire, UK

**Keywords:** Echocardiography, Emergency department, Ischemia, Myocardial infarction, POCUS, Point-of-care, Ultrasound

## Abstract

**Background:**

Chest pain is responsible for millions of visits to the emergency department (ED) annually. Cardiac ultrasound can detect ischemic changes, but varying accuracy estimates have been reported in previous studies. We synthetized the available evidence to yield more precise estimates of the accuracy of cardiac ultrasound for acute myocardial ischemia in patients with chest pain in the ED and to assess the effect of different clinical characteristics on test accuracy.

**Methods:**

A systematic search for studies assessing the diagnostic accuracy of cardiac ultrasound for myocardial ischemia in the ED was conducted in MEDLINE, EMBASE, CENTRAL, CINAHL, LILACS, Web of Science, two trial registries and supplementary methods, from inception to December 6th, 2022. Prospective cohort, cross-sectional, case–control studies and randomized controlled trials (RCTs) that included data on diagnostic accuracy were included. Risk of bias was assessed with the QUADAS-2 tool and a bivariate hierarchical model was used for meta-analysis with paired Forest and SROC plots used to present the results. Subgroup analyses was conducted on clinically relevant factors.

**Results:**

Twenty-nine studies were included, with 5043 patients. The overall summary sensitivity was 79.3% (95%CI 69.0–86.8%) and specificity was 87.3% (95%CI 79.9–92.2%), with substantial heterogeneity. Subgroup analyses showed increased sensitivity in studies where ultrasound was conducted at ED admission and increased specificity in studies that excluded patients with previous heart disease, when the target condition was acute coronary syndrome, or when final chart review was used as the reference standard. There was very low certainty in the results based on serious risk of bias and indirectness in most studies.

**Conclusions:**

Cardiac ultrasound may have a potential role in the diagnostic pathway of myocardial ischemia in the ED; however, a pooled accuracy must be interpreted cautiously given substantial heterogeneity and that important patient and test characteristics affect its diagnostic performance.

*Protocol Registration*: PROSPERO (CRD42023392058).

**Supplementary Information:**

The online version contains supplementary material available at 10.1186/s13049-024-01192-3.

## Background

Chest pain is one of the leading causes for emergency department (ED) visits among adults with more than one million cases reported in England's emergency services during the 2021–2022 period [1] and more than 7 million ED visits in the United States in 2020, corresponding to approximately 5.5–5.7% of all ED visits and to 20% of hospital admissions [1–3].

Chest pain can be caused by a wide range of diseases from life-threatening conditions such as acute coronary syndrome (ACS), aortic dissection, pulmonary embolism or pericardial effusion, to more benign and harmless entities [4, 5], making it a diagnostic challenge for emergency physicians. Ultimately, only 5.1% of all chest pain visits are diagnosed with acute coronary syndrome [5], but a missed diagnosis can be clinically devastating with high impact on morbidity and mortality [6, 7].

Transthoracic echocardiography (TTE) has been traditionally performed by cardiologists to provide comprehensive information on structural, functional, and hemodynamic aspects of the heart and great vessels and requires an extensive knowledge base with training standards [8–10]. The development of smaller and portable devices along with ultrasound education, training and certification in different medical specialties has led to the emergence of cardiac ultrasound protocols for different clinical settings, and to other specialists taking care of patients with cardiovascular emergencies becoming proficient in cardiac ultrasound imaging [10–14].

Cardiac ultrasound is superior to physical examination in correctly identifying the majority of cardiovascular conditions and can provide and an early assessment of left and right ventricular function, regional wall motion abnormalities (RWMA), pericardial effusion or valvular disorders [15–18]. Furthermore, clinical examination assisted by a focused cardiac ultrasound (FoCUS) increases sensitivity by 41% compared to clinical examination alone for the diagnosis of left ventricular dysfunction and 25% for the detection of moderate to severe aortic and mitral valve disease [19, 20].

Changes in regional wall motion can be visualized by echocardiography within seconds of myocardial ischemia even before electrocardiographic changes, as shown in patients undergoing coronary angioplasty after balloon inflation [21, 22] RWMA can be seen during transient ischemia with normal cardiac markers [21, 23, 24], and the absence of RWMA in patients with on-going chest pain has been reported to have a high negative predictive value for acute myocardial ischemia [25], giving cardiac ultrasound a potential role in the early detection of acute coronary syndromes in patients with chest pain in the ED.

Several studies have assessed cardiac ultrasound diagnostic accuracy for acute myocardial ischemia in patients with acute chest pain with a wide range of sensitivities (21% to 100%) [26, 27] and specificities (33% to 100%) reported [28, 29]. This uncertainty may be due to the fact that many of these studies are small, and the patient spectrum, ultrasound timing, protocols, devices and providers vary [24, 25, 30–32]. The aim of this systematic review is to identify, evaluate and synthetize all the available evidence to yield more precise estimates of the accuracy of cardiac ultrasound for the diagnosis of acute myocardial ischemia in patients with chest pain in the ED and to assess how different patient populations, and clinical or ultrasound characteristics may impact test accuracy [33].

## Methods

This review was conducted according to the Cochrane Handbook for Systematic Reviews of Diagnostic Test Accuracy [34], and results reported according to the preferred reporting items for systematic review and meta-analysis of diagnostic test accuracy studies (PRISMA-DTA), included in e-Appendix 1 (Additional File 1) [35]. The protocol for this review was registered in PROSPERO (CRD42023392058).

### Search methods

Systematic searches were conducted in MEDLINE, EMBASE, CENTRAL, CINAHL and LILACS from the date of inception to December 6th, 2022, with no language restrictions, and no additional filters. Supplementary searches were conducted in the Web of Science Complete Core Collection (including the Science citation index and the Conference Proceedings citation index), two trial registries (ClinicalTrials.gov and International Clinical Trials Registry Platform), Google Scholar and Google (until 5 pages with no relevant results were retrieved) and finally, by checking reference lists of studies included in the review. Consultation with an experienced librarian was undertaken during the development and implementation of the search strategy [36]. Search terms included a combination of MeSH terms (Medical Subject Headings) and free text using permutations of the search terms “ultrasonography”, “myocardial ischemia” and “emergency” (see e-Appendix 2 in Additional File 1 for details).

### Eligibility criteria

The review included prospective cohort, cross-sectional, case–control studies and randomized controlled trials of interventions that included data on diagnostic accuracy. Case reports/series, animal studies, and retrospective studies were excluded [37, 38]. We included studies conducted in adults with chest pain or chest pain equivalents of any duration, alone or in combination with other signs or symptoms presenting to the ED or chest pain units within the ED. In cases of a mixed clinical presentation, studies that reported separate data for patients with chest pain and studies where the majority of patients presented with chest pain were included. Trauma, postcardiac arrest, and mechanically ventilated patients were excluded as well as prehospital, primary/ambulatory care, hospital wards, and intensive/coronary care unit settings. Index test was any cardiac ultrasound protocol including transthoracic echocardiography (comprehensive or limited) or point-of-care ultrasound (POCUS) protocols, assessing regional wall motion abnormalities or left ventricular decreased contractility, conducted at the patient’s bedside, performed by physicians, training physicians or sonographers with the intention of identifying acute myocardial ischemia or evaluating the etiology of chest pain. Studies using only speckle-tracking and strain echocardiography were excluded based on limited availability of this technology in emergency settings affecting generalizability of findings as well as ultrasounds performed by radiologists, nurses, or conducted inside a radiology suite. Studies that assessed any type of myocardial ischemia as target condition (ACS, myocardial infarction and/or unstable angina, significant coronary artery stenosis), using any test positivity criteria and using any relevant reference standard or combination of reference standards to diagnose acute myocardial ischemia were included. ACS was defined as the spectrum of disease including unstable angina, non-ST and ST-segment elevation myocardial infarction [39, 40]. Any definition of significant coronary artery stenosis was included. Studies that did not provide sufficient data to construct a 2 × 2 table were excluded.

### Screening, selection, and data extraction

Literature searches were uploaded into Covidence systematic review software, Veritas Health Innovation, Melbourne, Australia (available at www.covidence.org) and underwent de-duplication. Two independent review authors conducted title and abstract screening (VZ and RMN), full text review of all potentially relevant studies identified (VZ and MCAG) and data extraction (VZ and MCAG). Discrepancies were resolved by discussion and disagreements were resolved by referral to a third reviewer. Multiple reports from the same study were merged to avoid bias [41, 42] and additional information from authors was sought to resolve any questions regarding eligibility if needed. Data was extracted into a pre-defined data extraction template (e-Appendix 3).

### Assessment of methodological quality

Risk of bias and applicability was assessed by two independent reviewers (VZ and MCAG) using the QUADAS-2 tool [43, 44]and disagreements were resolved through consensus. A predefined quality assessment template was used.

### Data synthesis and statistical analyses

Data extracted from all studies was used to construct the two-by-two tables and individual study estimates of sensitivity and specificity were presented graphically in paired Forest plots. Meta-analysis was conducted using a bivariate hierarchical random-effects model [33, 45–47]. An overall summary point with 95% confidence region and 95% prediction region were estimated and displayed in a summary receiver operator characteristic (SROC) plot. Planned subgroup analyses were conducted to assess how diagnostic accuracy varied across different subgroups (ultrasound protocol, operator, device, timing of ultrasound, and reference standard) [48]. Exploratory analyses for other sources of heterogeneity were conducted by visual inspection of forest plots and summary points and confidence regions in SROC plots. Based on these exploratory analyses, additional post-hoc subgroup analyses were conducted for patient spectrum and target condition. Summary estimates of sensitivity and specificity for each subgroup were calculated, SROC plots with summary points and 95% confidence regions were obtained for the different subgroups [33, 45, 46] and the Likelihood Ratio test was used for hypothesis testing of statistical significance between subgroups [49, 50].

All statistical analyses were conducted using STATA 18.0 Basic Edition with the recently developed and validated Metadta command [49, 51] Deek’s funnel plot was constructed to assess for publication bias and other sample size related effects and Deek’s test was conducted to assess funnel plot asymmetry using midas command for STATA [47, 52] Sensitivity analyses were conducted removing studies judged to be at high risk of bias, studies with inappropriate reference standard and unpublished studies, respectively. The GRADE approach was used to assess the certainty of evidence and results displayed using GRADEpro GDT [53–55].

## Results

**Fig. 1:**
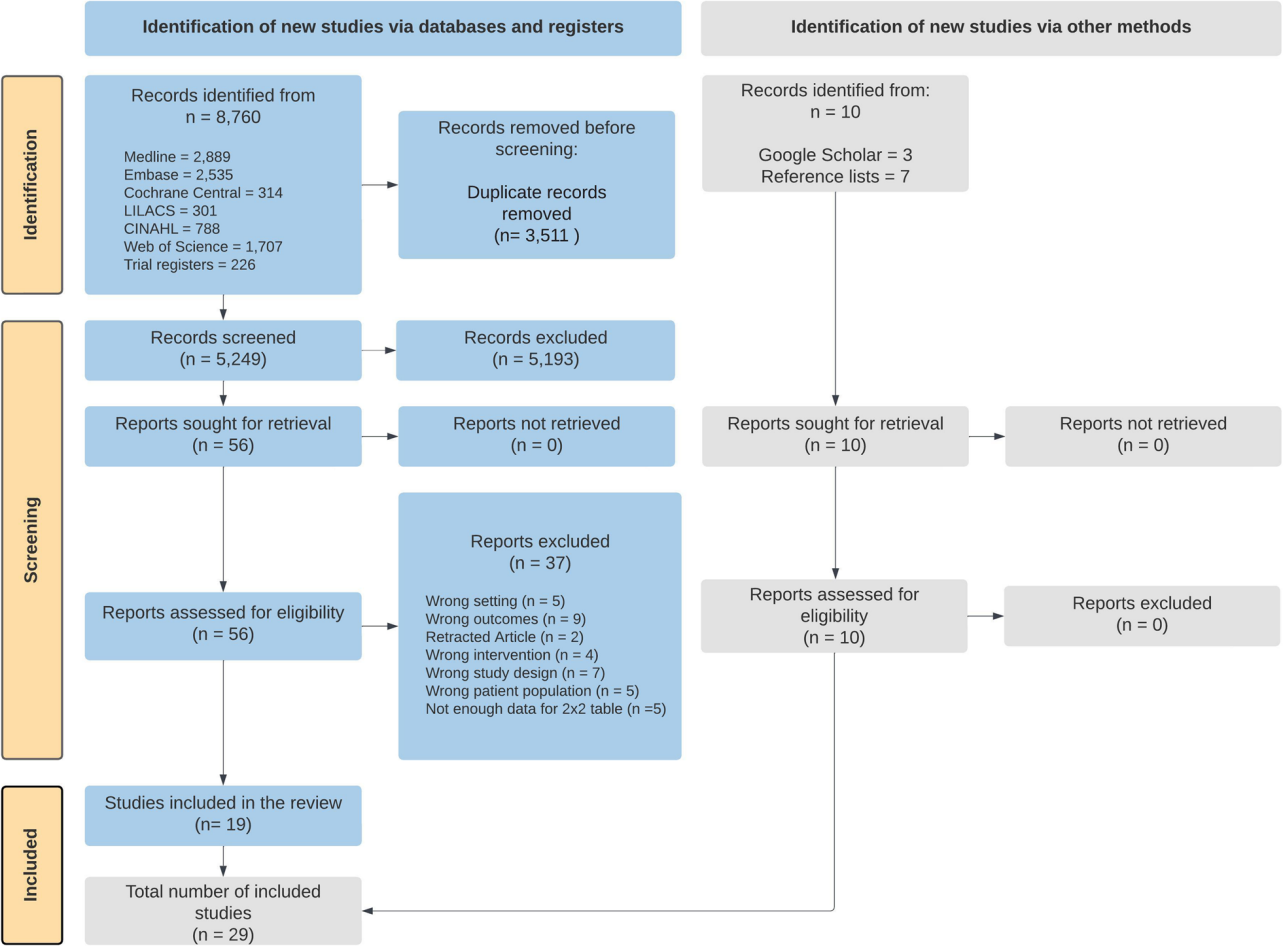
PRISMA flow diagram of search results and study selection

Searching in databases and registers identified 8760 studies. After removing duplicates and title and abstract screening, 56 full text studies were assessed for eligibility and 19 studies were identified. An additional 10 eligible studies were identified through search engines and handsearching of reference lists of included studies, with a total of 29 studies included in the review (Fig. 1) [24–30, 56–77].

### Characteristics of included studies

Key characteristics of the included studies are summarized in Table 1. Mean age of the population across studies ranged from 49 to 68 years (average: 59 years [SD 5.2]) and the proportion of male patients ranged from 42.3 to 84.5% (mean 62.6% [SD 11.9]). Patient spectrum varied across the studies, mainly in the inclusion (13 studies) [24, 29, 30, 56, 61, 63, 65, 70, 72–74, 76, 77] or exclusion (13 studies) [25, 27, 57–60, 62, 64, 66, 67, 69, 71, 75] of patients with previous heart disease. See e-Table 1 (Additional File 1: e-Appendix 4) for additional information.

**Table 1:** Description of included studies

Study ID	Country	Language	Study design	Recruitment Dates	Excluded Heart Disease	Total Patients	Age (years) Mean (SD)	Prevalence Disease	Target Condition	Reference Standard
Ahn [30]	Korea	English	Cohort study	Jan 2011–Dec 2011	No	308	67.7 (19.1)	22.1%	ACS	Final diagnosis
Allen [56]	United States	English	Cohort study	NA	No	178	59 (NA)	3.9%	MI	Not reported
Atar [76]	Israel	English	Cohort study	NA	No	70	50 (12)	14.3%	ACS	Chart Review
Bracey [28]	United States	English	Cohort study	NA	NR	16	61 (15.2)	81.3%	Significant Coronary Stenosis	Coronary Angiography
Castini [27]	Italy	Italian	Cohort study	NA	Yes	22	63 (9)	31.8%	Myocardial Infarction	Combination of Tests
Cevrim [57]	Turkey	English	Cohort study	Jun 2011–Dec 2011	Yes	48	49 (13)	8.3%	ACS	Final diagnosis
Chandra [77]	United States	English	Cohort study	Dec 2014	No	26	NA	15.4%	ACS	Chart Review
Dahlslett [58]	Norway	English	Cohort study	NA	Yes	64	CAD + : 54 (12) CAD-: 56 (12)	45.3%	Significant Coronary Stenosis	Coronary Angiography
DeLoizaga [59]	United States	English	Cohort study	Dec 2012–Nov 2013	Yes	80	56 (13)	10.0%	ACS	Chart Review
DiPasquale [23]	Italy	English	Cohort study	Dec 2000–Feb 2002	Yes	280	59.7 (11.9)	65.0%	Significant Coronary Stenosis	Coronary Angiography
Hickman [61]	UK	English	Cohort study	NA	No	80	64 (13.5)	16.3%	MI	Cardiac Enzymes
Kang [62]	Korea	English	Cohort study	Oct 2001–Dec 2002	Yes	114	60 (10)	76.3%	ACS	Combination of Tests
Kontos [63]	United States	English	Cohort study	Aug 1994–Dec 1994	No	260	54 (14)	17.3%	MI	Combination of Tests
Korosoglou [64]	Germany	English	Cohort study	NA	Yes	98	ACS + : 65 (12) ACS-: 57 (15)	37.8%	ACS	Chart Review by Independent person
Kountana [65]	Greece	English	Cohort study	Mar 2010–Dec 2011	No	33	59.8 (10.8)	15.2%	ACS	Combination of Tests
Lee [66]	Korea	English	Cohort study	Jan 2011–Dec 2011	Yes	69	55 (14)	36.2%	Significant Coronary Stenosis	Coronary Angiography
Mahmoud [24]	Saudi Arabia	English	Cohort study	Aug 2014–Oct 2016	No	250	67 (2.4)	53.2%	ACS	Chart Review
Mohler [29]	United States	English	Cohort study	NA	No	100	55.6 (12)	59.8%	ACS	Chart Review
Oh [67]	United States	English	Cohort study	NA	Yes	26	58 (NA)	26.9%	MI	Combination of Tests
Onishi [68]	Japan	English	Cohort study	NA	NR	91	NA	50.5%	ACS	Coronary Angiography
Peels [69]	Netherlands	English	Cohort study	Dec 1987–Nov 1988	Yes	43	54 (NA)	58.1%	Significant Coronary Stenosis	Coronary Angiography
Sabia [70]	United States	English	Cohort study	Apr 1987–Jun 1987	No	180	62 (14)	17.2%	MI	Cardiac Enzymes
Santana [26]	Brazil	Portuguese	Cohort study	Jun 2006–Jun 2008	NR	71	56 (NA)	30.6%	ACS	Final diagnosis
Sasaki [25]	United States	English	Cohort study	NA	Yes	46	60 (11)	50.0%	ACS	Combination of Tests
Shiran [71]	Israel	English	Cohort study	NA	Yes	605	58 v	12.2%	ACS	Final diagnosis
Sobczyk [72]	Poland	English	Cohort study	Jan 2013–Jan 2014	No	1312	67 (12.3)	79.2%	MI	Chart Review
Swinburn [73]	United Kingdom	English	Cohort study	NA	No	80	64 (13.5)	15.0%	MI	Combination of Tests
Weston [74]	United States	English	Cohort study	NA	No	108	HHE + : 58 (13), HHE -: 52 (12)	11.1%	ACS	Combination of Tests
Wilben [75]	India	English	Cross sectional study	Nov 2017–Oct 2019	Yes	385	55.1 (14.2)	81.0%	ACS	Coronary Angiography

SD Standard Deviation, NA Not Available, EKG Electrocardiogram, CAD Coronary Artery Disease, HHE Hand-Held Echocardiography, PTCA Percutaneous Transluminal Coronary Angioplasty, CABG Coronary Artery Bypass Graft, MRI Magnetic Resonance Imaging, CCTA Coronary Computed Tomography Angiography, ACS Acute Coronary Syndrome, MI Myocardial Infarction,

Cardiac ultrasound was conducted as POCUS in 5 studies [28, 30, 59, 72, 74] and as a TTE in 20 studies (either standard [24, 26, 29, 60–62, 66, 67, 70, 71, 73, 75, 76] or limited-TTE).[25, 27, 57, 58, 63, 64, 69] Cardiac ultrasound operators were from the cardiology department in 6 studies (cardiologists, cardiology fellows or sonographers),[27, 60, 63, 66, 67, 76] emergency department in 6 studies (emergency physicians or residents),[28, 30, 57, 59, 72, 77] sonographers without a description of department or training backgrounds in 3 studies[24, 29, 75], and not reported in 11 studies.[25, 26, 56, 58, 62, 64, 65, 68–71] In two studies, the person performing the ultrasound was described as “an experienced operator” [61, 73] and in one study was performed by 3rd year medical students [74]. The timing of cardiac ultrasound varied widely across all studies. Of note, nine studies reported ultrasound was conducted at arrival to the ED or immediately after arrival [24, 29, 60, 69, 70, 72, 73, 75, 76], during the first 4 h[63], 8 h [25], 12 h [62],or 24 h [71], and in most studies [16] the timing of ultrasound was not reported [27, 28, 30, 56–59, 61, 64–68, 74, 77]. Full echocardiography devices were used in 69% of studies [20], hand-held or cart/tray based devices in four studies [28, 57, 74, 76] and not reported in five studies [26, 56, 59, 65, 77]. The characteristics of the index test for the included studies are summarized in Table 2.

**Table 2:** Characteristics of the included studies: index test

Study ID	US Device	US Exam	US Operator	US Time	Positive Index Test
Ahn [30]	Echocardiography Device	POCUS	EM physician; EM resident	Not reported	RWMA only
Allen [56]	Not Reported	Not Reported	Not Reported	Not reported	Not Reported
Atar [76]	Handheld—Cart/Tray	TTE	Cardiologist	At admission	RWMA and/or LVEF reduction
Bracey [28]	Handheld—Cart/Tray	POCUS	EM physician	Not reported	RWMA only
Castini [27]	Echocardiography Device	Limited TTE	Cardiologist	Not reported	RWMA only
Cevrim [57]	Handheld—Cart/Tray	Limited TTE	EM physician	Not reported	RWMA only
Chandra [77]	Not Reported	Not Reported	EM physician	Not reported	RWMA and/or LVEF reduction
Dahlslett [58]	Echocardiography Device	Limited TTE	Not Reported	Not reported	RWMA only
DeLoizaga [59]	Not Reported	POCUS	EM physician	Not reported	RWMA only
DiPasquale [23]	Echocardiography Device	TTE	Cardiologist; Cardiology fellow	At admission	RWMA only
Hickman [61]	Echocardiography Device	TTE	Other: Experienced Operator	Not reported	RWMA only
Kang [62]	Echocardiography Device	TTE	Not Reported	< 12 h	RWMA only
Kontos [63]	Echocardiography Device	Limited TTE	Cardiology fellow; Sonographer	< 4 h	RWMA and/or LVEF reduction
Korosoglou [64]	Echocardiography Device	Limited TTE	Not Reported	Not reported	RWMA only
Kountana [65]	Not Reported	Not Reported	Not Reported	Not reported	RWMA only
Lee [66]	Echocardiography Device	TTE	Cardiology; sonographer	Not reported	RWMA only
Mahmoud [24]	Echocardiography Device	TTE	Sonographer	At admission	RWMA and/or LVEF reduction
Mohler [29]	Echocardiography Device	TTE	Sonographer	At admission	RWMA only
Oh [67]	Echocardiography Device	TTE	Cardiologist	Not reported	RWMA only
Onishi [68]	Echocardiography Device	Not Reported	Not Reported	Not reported	RWMA only
Peels [69]	Echocardiography Device	Limited TTE	Not Reported	At admission	RWMA only
Sabia [70]	Echocardiography Device	TTE	Not Reported	At admission	RWMA only
Santana [26]	Not Reported	TTE	Not Reported	Not reported	RWMA only
Sasaki [25]	Echocardiography Device	Limited TTE	Not Reported	< 8 h	RWMA only
Shiran [71]	Echocardiography Device	TTE	Not Reported	< 24 h	RWMA only
Sobczyk [72]	Echocardiography Device	POCUS	EM resident	At admission	RWMA only
Swinburn [73]	Echocardiography Device	TTE	Other: Experienced Operator	At admission	RWMA only
Weston [74]	Handheld—Cart/Tray	POCUS	Other: 3rd year medical student	Not reported	RWMA and/or LVEF reduction
Wilben [75]	Echocardiography Device	TTE	Sonographer	At admission	RWMA only

US Ultrasound, TTE Transthoracic Echocardiography, POCUS point-of-care ultrasound, RWMA Regional Wall Motion Abnormalities, LVEF Left ventricle ejection fraction

The reference standard varied widely across studies, using final diagnosis or chart review [26, 29, 30, 57, 59, 64, 71, 72, 76, 77], cardiac enzymes results [61, 70, 73], a combination of tests (between ECG, cardiac enzymes, non-invasive testing, and coronary angiography) [24, 25, 27, 62, 63, 65–67, 74] or significant coronary artery stenosis [28, 58, 60, 68, 69, 75]. The definition of significant coronary stenosis varied between ≥ 50% [58, 69] to ≥ 70% narrowing in epicardial coronary arteries [23, 66]. It is important to mention that cardiac enzymes varied across time, with studies conducted in the 1990’s and early 2000’s using CK and CKMB and studies from the late 2000’s onwards using troponin and high sensitivity troponin.

### Quality assessment of included studies.

Overall, most studies were at high risk of bias due to patient selection and reference standard factors. High risk of bias was found in the patient selection domain (22 studies, 76%) [24–29, 56–60, 62–67, 69–72, 74, 75] due to convenience sampling or inappropriate exclusions (patients with previous heart disease, low or high risk of ACS and/or inadequate ultrasound windows); in the reference standard domain (15 studies, 52%) [24, 26, 27, 29, 57, 61, 63, 66–68, 70–74] due to lack of blinding or because it was not likely to correctly classify myocardial ischemia [24, 26, 27, 29, 57, 61, 63, 66–68, 70–74]; and in the flow and timing (13 studies, 45%) [24–26, 28, 29, 57, 66, 68–72, 74] due to partial or differential verification or the exclusion of patients from the analysis (i.e., patients with inadequate echocardiographic windows) [24–26, 28, 29, 57, 66, 68, 70, 71, 74]. The index test domain was judged to be at low risk of bias in most studies (28 studies, 97%).

**Fig. 2:**
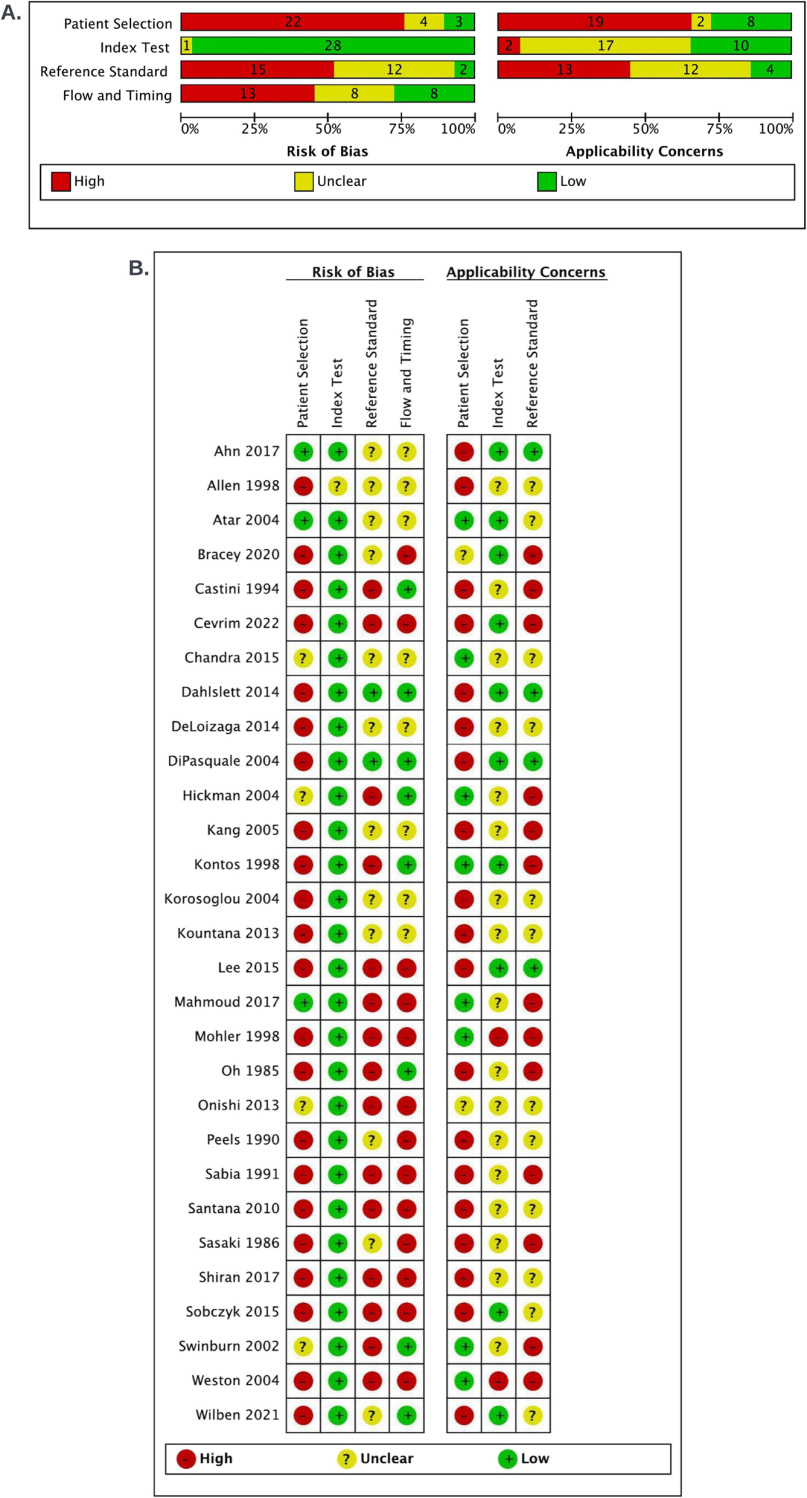
Risk of bias and applicability concerns graph (**A**) and summary table (**B**): Review author’s judgements about each domain are presented as percentages across included studies and for each included study

Nineteen studies (65%) [25–27, 30, 56–60, 62, 64–67, 69–72, 75], were judged to have high concerns of applicability for patient selection due to the inappropriate exclusion of patients (either previous heart disease or low/high probability of coronary disease) limiting the applicability of the findings to patients commonly presenting to the ED with chest pain. Thirteen studies (45%) were judged to have high concerns of applicability in the reference standard domain [24, 25, 27–29, 57, 61–63, 67, 70, 73, 74] due to the use of CK, CK-MB and LDH as cardiac biomarkers, which are not currently used in the diagnostic pathway of myocardial ischemia and in some studies, the definition of myocardial ischemia as target condition does not match the current definition [24, 25, 27–29, 57, 61–63, 67, 70, 73, 74]. Index test was found to have uncertain (16 studies) [24–27, 56, 59, 61, 62, 64, 65, 67–71, 73, 77] or low concerns (11 studies), [23, 28, 30, 57, 58, 63, 66, 72, 75, 76] and two studies with high concerns for applicability.[29, 74] (Fig. 2 and e-Appendix 5 in Additional File 1).

### Summary of findings

**Fig. 3:**
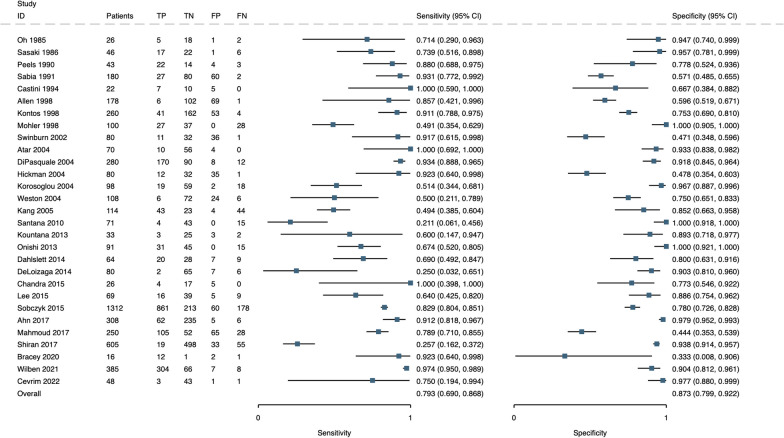
Paired Forest plot of sensitivity and specificity of all included studies. TP = true positive, TN = true negative, FP = false positive, FN = false negative

There was substantial heterogeneity across the 29 studies, with sensitivities ranging from 21%[26] to 100%[77] and specificities from 33%[28] to 100%[29] (Fig. 3).

Results are displayed in chronological order to assess possible trends with technological developments or diagnostic criteria over time, however, no clear trend was identified across time according to the year of publication [78].

**Fig. 4:**
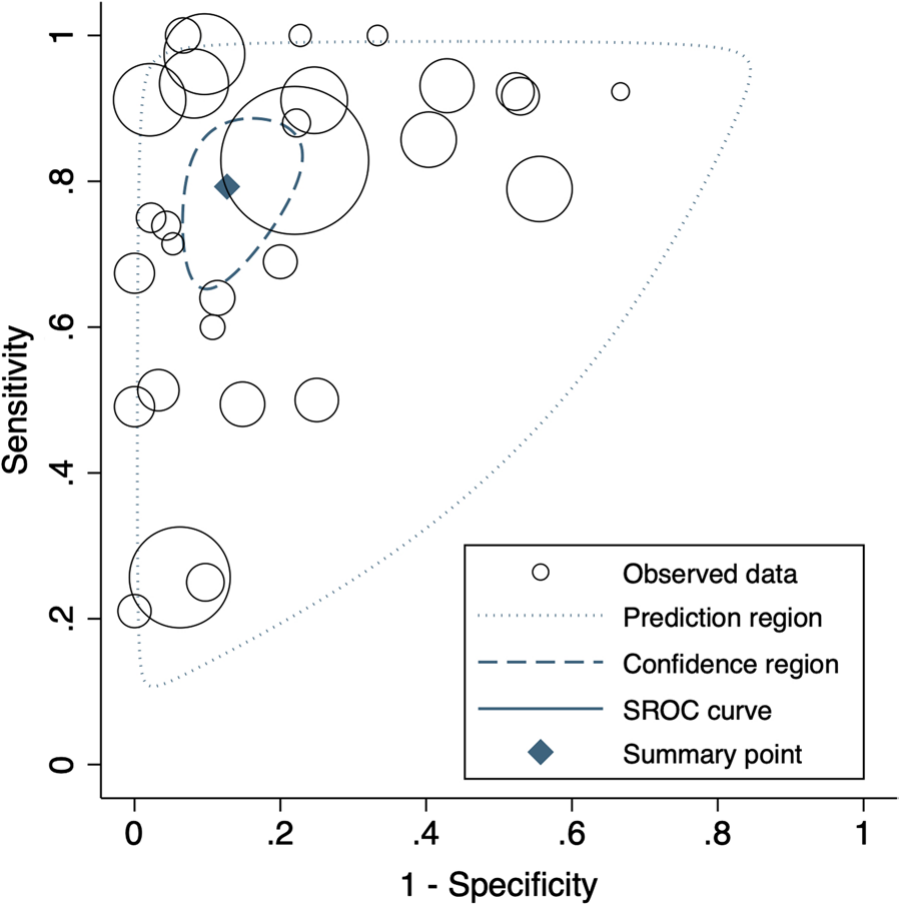
SROC plot of sensitivity and specificity of all included studies. Each study is represented by a circle, with the size of the circle indicating the size of the sample

The median proportion of patients with the target condition across studies was 26.9% (IQR 15.2—53.2%). The summary estimate of sensitivity and specificity was 79.3% (95%CI 69.0 to 86.8%) and 87.3% (95%CI 79.9 to 92.2%), respectively, with a wide 95% predictive region in the SROC plot (Fig. 4). Based on GRADE, the overall assessment of certainty was very low based on very serious risk of bias, and indirectness and serious imprecision (Table 3). More detailed explanations on each domain grading are provided in e-Table 2 (Additional File 1e-Appendix 6). [53–55].

**Table 3:** Summary of findings

Review question: What is the diagnostic accuracy of cardiac ultrasound for the diagnosis of myocardial ischemia in patients with chest pain in the ED?
*Population***:** Adults patients with chest pain. Some studies excluded patients with previous HD or low/high risk of coronary disease
*Setting*: Emergency Department
*Study design*: Prospective cohort studies, cross-sectional
*Index test*: Cardiac ultrasound (TTE, Limited TTE, POCUS)
*Target Condition*: Any Myocardial Ischemia (Myocardial infarction, ACS, Critical coronary Stenosis)
*Reference Standards*: Any reference standard (Final chart review, combination of tests, coronary angiogram or cardiac enzymes)
*Limitations in the evidence*
High risk of bias and applicability concerns, mainly driven by patient selection (exclusion of patients with HD, low or high risk of myocardial ischemia and patients with difficult ultrasound window) and by reference standard (lack of blinding and reference standard not correctly classifying the disease)
• Patient selection: High or unclear risk of bias in 26 (90%) studies; high or unclear concern of applicability in 21 (72%) studies
• Index test: Low risk of bias in 28 (97%) of studies, one with unclear risk of bias; high or unclear concern of applicability in 19 (66%) studies
• Reference Standard: High or unclear risk of bias in 27 (93%) studies; high or unclear concern of applicability in 25 (86%) studies
• Flow and timing: High or unclear risk of bias in 21 (72%) studies
Overall assessment: Most studies were at risk of bias (90%) and had concerns regarding applicability (90%)

Findings				
Numer of studies (Participants)	Median proportion with target condition % (IQR)	Summary sensitivity % (95% confidence interval)	Summary specificity % (95% confidence interval)	GRADE Certainty of Evidence^a^
29 5043	26.9 (15.2–53.2)	79.3 (69.0–86.8)	87.3 (79.9–92.2)	⨁◯◯◯ Very low

In a virtual population of 1000 patients with chest pain in the ED, assuming a prevalence of 27%, 270 patients will have acute myocardial ischemia. Of these, cardiac ultrasound will detect 214 patients with myocardial ischemia, but 56 patients will be missed (false negatives). For the 730 patients without the target condition, 93 patients will wronly diagnosed with myocardial ischemia (false positives)

ED Emergency department; TTE trasnthoracic echocardiography; ACS acute coronary syndrome; EKG electrocardiogram; IQR Interquartile range, HD Heart Disease

^a^ GRADE approach was used for the assessment of certainty of evidence: Risk of bias was rated very serious, because 90% of studies had a high risk of bias in one or more QUADAS-2 domains, indirectness was rated very serious because 90% of studies had high concerns of applicability in one or more QUADAS-2 domains, inconsistency was rated not serious becasue substantial heterogeneity was explained by patient spectrum, timing of ultrasound reference standard and target condition, imprecision was rated serious for sensitiviy and not serious for specificity. No publication bias was detected

### Investigation of heterogeneity and subgroup analyses

Subgroup analyses were conducted to identify sources of heterogeneity and to assess the effect of specific study characteristics on the diagnostic accuracy of cardiac ultrasound in patients admitted with chest pain to the ED. Table 4 describes subgroup analyses for key study characteristics. Forest plots and a detailed description of all subgroup analyses are included in e-Appendix 7 (Additional File 1).

**Table 4:** Diagnostic Accuracy of cardiac ultrasound—Subgroup Analyses

	Number of studies (Participants)	Number of patients with Myocardial Ischemia (%)	Summary sensitivity % (95% confidence interval)	*P* value	Summary specificity % (95% confidence interval)	*P* value
Patient spectrum						
Excludes HD	13 (1880)	820 (43.6)	74.1 (58.0 to 85.5)	0.168	91.0 (84.1 to 95.1)	0.029
Includes HD	13 (2985)	1432 (48.0)	85.8 (73.5 to 93.0)		78.0 (66.1 to 86.6)	
Ultrasound Operator						
Cardiology	6 (727)	276 (38.0)	90.2 (73.7 to 96.8)	0.720	88.1 (71.8 to 95.5)	0.983
Emergency medicine	6 (1790)	1136 (63.5)	83.1 (60.2 to 94.1)		89.2 (73.4 to 96.1)	
Sonographer	3 (735)	500 (68.0)	83.7 (55.8 to 95.4)		87.6 (59.5 to 97.1)	
Timing of Ultrasound						
At admission	9 (2700)	1797 (66.6)	89.5 (78.9 to 95.1)	0.038	81.5 (66.2 to 90.8)	0.389
First 24 h	4 (1025)	229 (22.3)	62.8 (34.6 to 84.3)		89.5 (71.4 to 96.7)	
Type of Ultrasound Device						
Echocardiography device	20 (4413)	2248 (50.9)	81.1 (70.7 to 88.5)	0.809	87.1 (77.8 to 92.9)	0.945
Cart/Tray-based or Handheld	4 (242)	39 (16.1)	84.0 (53.2 to 96.0)		86.5 (58.1 to 96.7)	
Type of Cardiac Ultrasound Protocol						
TTE	23 (3193)	1186 (37.1)	79.1 (67.5 to 87.3)	0.827	88.3 (79.9 to 93.4)	0.681
POCUS	5 (1824)	1140 (62.5)	76.4 (47.9 to 91.9)		84.7 (59.5 to 95.4)	
Reference Standard						
Cardiac enzymes only	3 (340)	54 (15.9)	93.8 (73.9 to 98.8)	0.082	50.7 (23.8 to 77.3)	0.014
Chart review	11 (2968)	1451 (48.9)	65.8 (47.9 to 80.1)		93.0 (86.7 to 96.4)	
Combination of tests	7 (609)	186 (30.5)	74.2 (52.3 to 88.3)		85.2 (70.6 to 93.2)	
Coronary angiography	7 ( 948)	632 (66.7)	87.0 (73.2 to 94.3)		88.7 (76.2 to 95)	
Target Condition						
Myocardial infarction	8 (2138)	1159 (54.2)	90.7 (78.3 to 96.3)	0.035	67.4 (49.4 to 81.4)	0.002
Acute coronary syndrome	16 (2433)	897 (36.9)	68.0 (53.3 to 79.8)		93.2 (88.2 to 96.2)	
Significant coronary stenosis	5 (472)	274 (58.1)	85.0 (65.4 to 94.4)		82.7 (81.5 to 91.2)	

TTE Transthoracic Echocardiography, POCUS point-of-care ultrasonography, HD Heart Disease

**Fig. 5:**
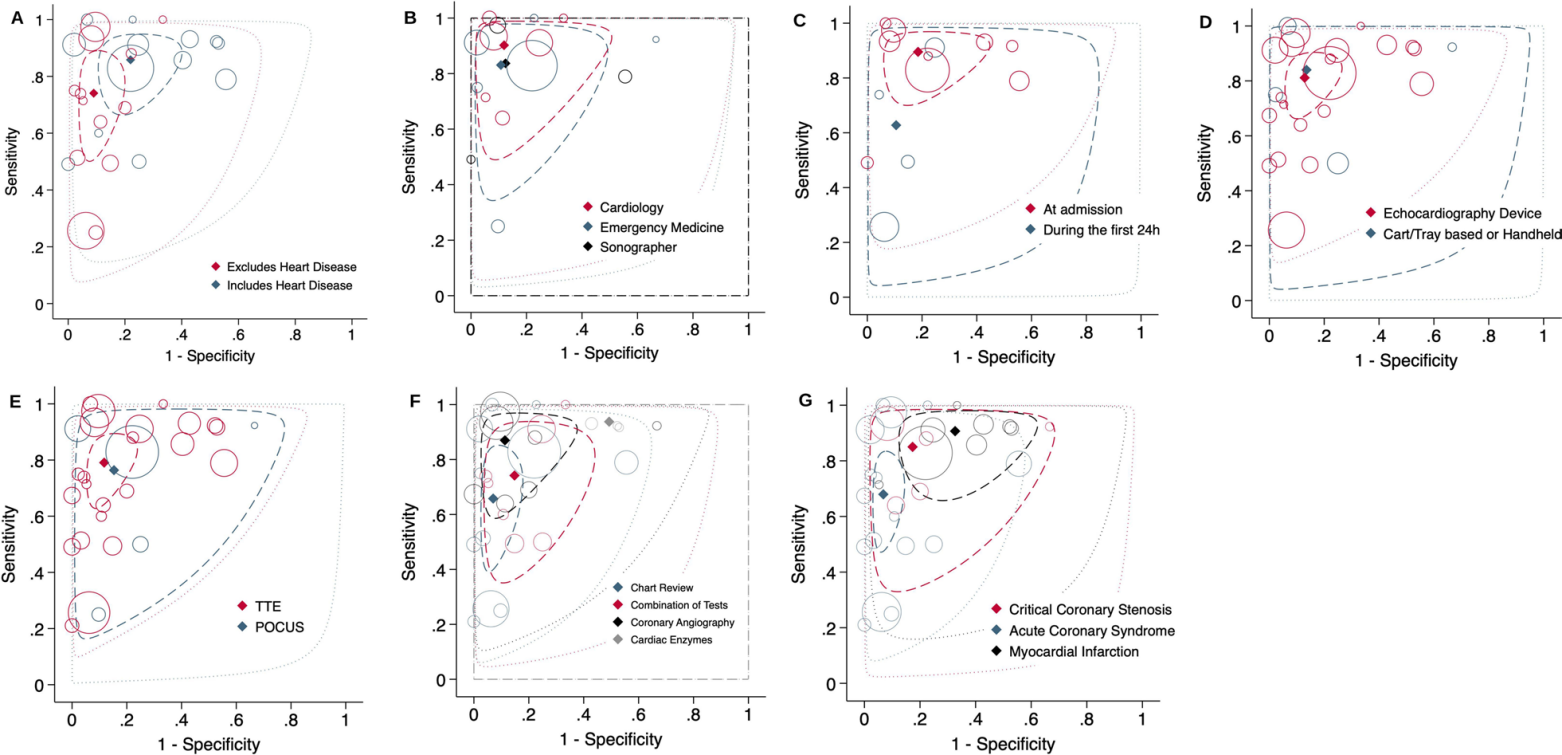
SROC plot of sensitivity and specificity in subgroup analyses, according to A = Patient Spectrum, B = Ultrasound Operator, C = Timing of Ultrasound, D = Ultrasound Device, E = Type of Ultrasound Protocol, F = Reference Standard, G = Target Condition. Diamond: summary estimate, dashed line: 95% confidence region, dotted line: 95% prediction region

Studies that excluded patients with previous heart disease showed a significantly higher specificity (91.0%, [95%CI 84.1 to 95.1%]) compared to studies that included patients with or without heart disease (78.0% [95%CI 66.1 to 86.6%], *P* = 0.029). Sensitivity was similar across both groups, with substantial heterogeneity between studies, as displayed by the wide prediction region (Fig. 5A).

There was no significant difference in sensitivity and specificity according to the ultrasound operator, the type of ultrasound device or the ultrasound scanning protocol used, as depicted in Fig. 5B, D and E respectively.

There was a significantly higher sensitivity in studies where ultrasound was conducted at admission or immediately after admission (89.5% [95%CI 78.9 to 95.1]), compared to studies where the timing of ultrasound was conducted at any time during the first 24 h (62.8% [95%CI 34.6 to 84.3, *p* = 0.038]) with similar specificity across both groups (*P* = 0.945) with substantial heterogeneity between studies in both groups represented in the wide 95% prediction region (Fig. 5C).

There was a significant difference in specificity across studies according to the reference standard used. Studies that used final chart review showed a high specificity (93.0% [95% CI 86.7 to 96.4%]), in contrast to studies that used cardiac enzymes only (50.7% [95% CI 23.8 to 77.3%]). Studies where the reference standard was coronary angiography, or a combination of tests had a specificity of 88.7% (95% CI 76.2 to 95%) and 85.2% (95% CI 70.6 to 93.2) respectively (Fig. 5F). Sensitivity was not significantly different across these groups.

The target condition also had a significant effect on sensitivity (*P* = 0.035) and specificity (*P* = 0.002) across groups (Fig. 5G). Pooled sensitivity was higher (90.7% [95%CI 78.3 to 96.3%]) in studies that used myocardial infarction as the target condition and lower in studies that used ACS (68.0%; 95%CI 53.3 to 79.8%). Pooled specificity was higher (93.2% [95%CI 88.2 to 96.2%]) for studies with ACS as the target condition and lower (67.4%; [95%CI 49.4 to 81.4%]) for myocardial infarction without an overlap between the 95% confidence regions.

Sensitivity analyses were conducted by removing studies with high risk of bias, studies with inappropriate reference standard and unpublished studies and did not show substantial changes in the effects estimates. A detailed description of these analyses is included in e-Appendix 8 (Additional File 1). Deek’s funnel plot for publication bias showed an acceptable symmetry with a *P* value of 0.91, suggesting no publication or small sample size study effect (Additional File 1: e-Fig. 15, e-Appendix 9).

## Discussion

This systematic review assessed the available evidence on cardiac ultrasound for the diagnosis of acute myocardial ischemia in patients with chest pain in the ED. Twentynine relevant studies were included in this review, with 5043 patients. The overall summary estimate for sensitivity and specificity was 79.3% (95%CI 69.0 to 86.8%) and 87.3% (95%CI 79.9 to 92.2%), respectively, but must be interpreted with caution given the substantial heterogeneity between studies.

Potential differences in patient spectrum, timing of ultrasound, reference standard and definition of the target condition suggested a significant effect on the performance of ultrasound. Studies that excluded patients with previous heart disease had a higher pooled specificity compared to studies that included patients with or without heart disease, with similar sensitivity, probably reflecting that excluding patients with previous RWMAs or reduced left ventricular ejection fraction may allow an easier identification of new RWMAs and decrease the number of false positives. Studies where cardiac ultrasound was conducted at admission or immediately after admission showed higher pooled sensitivity, compared to studies where the timing of ultrasound was conducted at any time during the first 24 h, most likely reflecting the transient nature of the myocardial RWMA during myocardial ischemia [21, 22].

The reference standard used significantly affected specificity of cardiac ultrasound, with a very low pooled specificity if only cardiac enzymes were used, compared to final chart review, coronary angiography or a combination of tests, probably because cardiac enzymes may also be elevated in myocardial injury of different etiologies not related to ischemia [79, 80]. Finally, the target condition also had a significant effect on sensitivity and specificity of cardiac ultrasound, showing a low pooled sensitivity in studies that assessed ACS (including unstable angina and myocardial infarction), likely reflecting the transient nature of RWMA in unstable angina. Studies that assessed myocardial infarction compared to the other target conditions had a low pooled specificity, since transient ischemic changes seen by ultrasound do not necessarily result in myocardial necrosis and infarction. The diagnostic accuracy of cardiac ultrasound was not significantly affected by the ultrasound operator, device or protocol used.

The diagnosis of acute myocardial ischemia in the ED is currently based on multiple criteria, with patients initially assessed according to history and physical examination, ECG findings, and high-sensitive troponins [5, 81]. Individually, these tests have limited diagnostic value. Clinical features and physical findings have sensitivities that range from 6.8% to 85.1% and specificities from 34.1% to 94.8% [82, 83]. The ECG has a very low sensitivity (29–45%) and a specificity from 67 to 94% [84, 85], and even ST-segment elevations have a false positive rate of 9% for coronary disease leading to unnecessary invasive procedures.[86] High-sensitivity troponin at admission has a sensitivity of 90% (95%CI 85–94%) and specificity of 78% (95%CI 72–83%), and multiple sampling protocols report a sensitivity and specificity of 99% (95%CI 98 to 100%) and 68% (95%CI 67 to 70%). A great limitation of high-sensitivity troponin is that it might be elevated in other disease states different than myocardial ischemia, like heart failure or chronic kidney disease and should not be used solely as a rule-in test [87]. Additionally, troponin laboratory turnaround time takes more than 90 min in most institutions in the United States [88] and frequently there is a need for a repeated sample at 1, 2 or 3 h, to avoid false negative results if blood samples are drawn too soon after the initiation of chest pain [87, 89]. Cardiac ultrasound can detect early regional myocardial wall motion abnormalities induced by ischemia [21, 22], and has been shown to be more accurate than history and physical exam for the detection of many cardiac conditions [18–20]. Early echocardiographic detection of myocardial ischemia may also help decrease physician’s diagnostic uncertainty [32, 90–92], improve patient’s outcomes by improving time to treatment [90], or shortening length of stay, and it could help reduce downstream testing and overall costs [18, 92, 93] by narrowing the differential diagnosis in patients with chest pain. Nevertheless, its role in the diagnostic pathway for myocardial ischemia in the ED is currently limited to a downstream test for uncertain cases. Thus, the first step to include cardiac ultrasound in this diagnostic pathway is to assess its accuracy and to understand the different clinical or technical factors that may affect its performance. This systematic review summarizes all available evidence, providing an overall diagnostic accuracy for cardiac ultrasound in patients with chest pain in the ED, which, in lieu of the individual diagnostic accuracy of currently used tests, may provide additional valuable information to the clinician to make a prompt and accurate diagnosis. Most importantly, this review provides an insight into the probable causes for the substantial heterogeneity, showing that the diagnostic performance of cardiac ultrasound may be significantly affected by different clinical and technical characteristics that will likely be encountered in the ED, where some patients admitted with chest pain will have previous heart disease, or some patients will be able to receive the cardiac ultrasound at admission, or later depending on training, equipment, and personnel availability. Therefore, an overall diagnostic accuracy must be interpreted carefully, and each specific factor must be considered for the clinician to assess the diagnostic value of the cardiac ultrasound in the individual patient. Most likely, a cardiac ultrasound performed at ED admission or in patients with on-going pain without previous heart disease would be the most clinically useful based on its diagnostic performance, with the additional potential of identifying and/or ruling out other possible causes of chest pain.

### Strengths and limitations

This review has several strengths and limitations. The large comprehensive search strategy is likely to have captured all relevant studies; a substantial number of studies and patients were included leading to greater precision in summary estimates, and a thorough evaluation of heterogeneity was based on clinical and physiological factors that are clinically relevant. Nevertheless, patient spectrum and target condition differences were found on post-hoc exploratory analysis. Some subgroups had a high degree of uncertainty in the estimates (i.e., timing of ultrasound during the first 24 h or cardiac enzymes reference standard) limiting the interpretation since the statistical power of the comparison also depends on the precision of the accuracy estimates. Individual patient data was not used for comparisons and uncontrolled bias from study-level comparisons and other confounders may have influenced the results. Significant differences found in these subgroup analyses are only exploratory in nature and may not reflect the true causal difference. Important temporal differences in the reference standard with the evolution of myocardial injury markers throughout the years may have influence the results, which was acknowledged in the quality assessment of the studies. However, there was no substantial temporal trend observed in the Forest Plot. Also, there was significant underreporting of important study characteristics and missing information that did not allow for a more robust assessment of its effect. There were only minor deviations from the protocol (Additional File 1: e-Table 7, e-Appendix 10).

Important consequences may arise from applying a cardiac ultrasound in the diagnostic pathway of patients with chest pain in the ED and significant uncertainty in the evidence from this review limits recommendations for clinical practice. Considering that ultrasound is a bedside, accessible and low-cost diagnostic test and that ultrasound training is becoming part of routine medical education in many countries, there is need for a well-designed diagnostic randomized trial that clarifies the potential role of cardiac ultrasound in the diagnostic pathway of patients with chest pain, ideally powered to assess differences according to patient’s previous heart disease, considering the complete spectrum of myocardial ischemia (unstable angina as well as myocardial infarction), and providing evidence of its effect on relevant patient clinical outcomes, resource-use outcomes and costs [94].

## Conclusion

Cardiac ultrasound may have a potential role in the diagnostic pathway of myocardial ischemia in the emergency department; however, the overall pooled accuracy must be interpreted cautiously given substantial heterogeneity and that important patient and test characteristics affect its diagnostic performance in this clinical context. Further well-designed research that clarifies its role in the diagnostic pathway and its clinical utility as an adjunct to clinical evaluation, ECG, and troponins for patients with chest pain in the emergency department needs to be conducted.

### Supplementary Information

**Additional file 1**. e-Appendix 2: Search Strategy.

## Data Availability

The datasets used and/or analysed during the current study are available from the corresponding author on reasonable request.

## References

[1] Sweeney M, Bleeze G, Storey S, Cairns A, Taylor A, Holmes C (2020). The impact of an acute chest pain pathway on the investigation and management of cardiac chest pain. Future Healthc J.

[2] Cairns CKK. National Hospital Ambulatory Medical Care Survey: 2020 emergency department summary tables.

[3] Secondary Care Analytical Team NHS Digital Hospital Accident & Emergency Activity 2021-22Sept 18, 2022. Available from: https://digital.nhs.uk/data-and-information/publications/statistical/hospital-accident--emergency-activity/2021-22.

[4] Stepinska J, Lettino M, Ahrens I, Bueno H, Garcia-Castrillo L, Khoury A, et al. Diagnosis and risk stratification of chest pain patients in the emergency department: focus on acute coronary syndromes. A position paper of the Acute Cardiovascular Care Association. Eur Heart J Acute Cardiovasc Care. 2020;9(1):76–89. 10.1177/2048872619885346 31958018

[5] Gulati M, Levy PD, Mukherjee D, Amsterdam E, Bhatt DL, Birtcher KK (2021). 2021 AHA/ACC/ASE/CHEST/SAEM/SCCT/SCMR guideline for the evaluation and diagnosis of chest pain: a report of the American college of cardiology/American heart association joint committee on clinical practice guidelines. Circulation.

[6] Kwok CS, Mallen CD (2021). Missed acute myocardial infarction: an underrecognized problem that contributes to poor patient outcomes. Coron Artery Dis.

[7] Pope JH, Aufderheide TP, Ruthazer R, Woolard RH, Feldman JA, Beshansky JR (2000). Missed diagnoses of acute cardiac ischemia in the emergency department. N Engl J Med.

[8] Wiegers SE, Ryan T, Arrighi JA, Brown SM, Canaday B, Damp JB, et al. 2019 ACC/AHA/ASE Advanced training statement on echocardiography (Revision of the 2003 ACC/AHA clinical competence statement on echocardiography): a report of the ACC competency management committee. circulation: cardiovascular imaging. 2019;12(7):e000026. 10.1002/ccd.28313 31313449

[9] Popescu BA, Andrade MJ, Badano LP, Fox KF, Flachskampf FA, Lancellotti P (2009). European association of echocardiography recommendations for training, competence, and quality improvement in echocardiography. Eur J Echocardiogr.

[10] Popescu BA, Stefanidis A, Fox KF, Cosyns B, Delgado V, Di Salvo GD (2020). Training, competence, and quality improvement in echocardiography: the European association of cardiovascular imaging recommendations: update 2020. Eur Heart J Cardiovasc Imaging.

[11] Expert Round Table on Echocardiography in ICU (2014). International consensus statement on training standards for advanced critical care echocardiography. Intensive Care Med.

[12] Mayo PH, Koenig S (2020). Advanced critical care echocardiography certification: an update. Chest.

[13] Nanjayya VB, Orde S, Hilton A, Yang Y, Costello C, Evans J, et al. Levels of training in critical care echocardiography in adults. Recommendations from the College of Intensive Care Medicine Ultrasound Special Interest Group. Australas J Ultrasound Med. 2019;22(1):73–79. 10.1002/ajum.12127 PMC8411793 34760542

[14] Cahalan MK, Stewart W, Pearlman A, Goldman M, Sears-Rogan P, Abel M (2002). American society of echocardiography and society of cardiovascular anesthesiologists task force guidelines for training in perioperative echocardiography. J Am Soc Echocardiogr.

[15] Spencer KT, Flachskampf FA (2019). Focused cardiac ultrasonography. JACC Cardiovasc Imaging.

[16] Levitov A, Frankel HL, Blaivas M, Kirkpatrick AW, Su E, Evans D (2016). Guidelines for the appropriate use of bedside general and cardiac ultrasonography in the evaluation of critically Ill patients-part II: cardiac ultrasonography. Crit Care Med.

[17] Marbach JA, Almufleh A, Di Santo P, Simard T, Jung R, Diemer G (2020). A shifting paradigm: the role of focused cardiac ultrasound in bedside patient assessment. Chest.

[18] Mehta M, Jacobson T, Peters D, Le E, Chadderdon S, Allen AJ (2014). Handheld ultrasound versus physical examination in patients referred for transthoracic echocardiography for a suspected cardiac condition. JACC Cardiovasc Imaging.

[19] Marbach JA, Almufleh A, Di Santo P, Jung R, Simard T, McInnes M (2019). Comparative accuracy of focused cardiac ultrasonography and clinical examination for left ventricular dysfunction and valvular heart disease: a systematic review and meta-analysis. Ann Intern Med.

[20] Stanger D, Wan D, Moghaddam N, Elahi N, Argulian E, Narula J, Ahmadi A. Insonation versus Auscultation in Valvular Disorders: Is aortic stenosis the exception? A systematic review. Ann Glob Health. 2019;85(1). 10.5334/aogh.2489 PMC6634326 31298821

[21] Hauser AM, Gangadharan V, Ramos RG, Gordon S, Timmis GC (1985). Sequence of mechanical, electrocardiographic and clinical effects of repeated coronary artery occlusion in human beings: echocardiographic observations during coronary angioplasty. J Am Coll Cardiol.

[22] Nesto RW, Kowalchuk GJ (1987). The ischemic cascade: temporal sequence of hemodynamic, electrocardiographic and symptomatic expressions of ischemia. Am J Cardiol.

[23] Di Pasquale P, Cannizzaro S, Scalzo S, Maringhini G, Sarullo FM, Cacia A, Paterna S (2004). Sensitivity, specificity and predictive value of the echocardiography and troponin-T test combination in patients with non-ST elevation acute coronary syndromes. Int J Cardiovasc Imaging.

[24] Mahmoud MZ (2017). Echocardiography in the evaluation of chest pain in the emergency department. Pol J Radiol.

[25] Sasaki H, Charuzi Y, Beeder C, Sugiki Y, Lew AS (1986). Utility of echocardiography for the early assessment of patients with nondiagnostic chest pain. Am Heart J.

[26] Santana G, Castro M, Luiz D, Gomes M, Andrade N, Natividade J (2010). Papel do Ecocardiograma Transtorácico de Rotina na Unidade de Dor Torácica. Rev bras ecocardiogr imagem cardiovasc.

[27] Castini D, Gentile F, Ornaghi M, Esposti D, Lippolis A, Mangiarotti E, Maggi GC. Utilità dell’ecocardiografia in pronto soccorso per la diagnosi precoce di infarto miocardico acuto. Il Cuore.11(3):243–50.

[28] Bracey AMM, Massey L, Singer AJ, Alian A, Secko M. Point-of-care ultrasound detects regional wall motion abnormalities and acute coronary occlusions. Academic Emergency Med. 2020;27:86.

[29] Mohler ER, Ryan T, Segar DS, Sawada SG, Sonel AF, Perkins L (1998). Clinical utility of troponin T levels and echocardiography in the emergency department. Am Heart J.

[30] Ahn JH, Jeon J, Toh HC, Noble VE, Kim JS, Kim YS (2017). SEARCH 8Es: a novel point of care ultrasound protocol for patients with chest pain, dyspnea or symptomatic hypotension in the emergency department. PLoS ONE.

[31] Kontos MC (1999). Role of echocardiography in the emergency department for identifying patients with myocardial infarction and ischemia. Echocardiography.

[32] Buhumaid RE, St-Cyr Bourque J, Shokoohi H, Ma IWY, Longacre M, Liteplo AS (2019). Integrating point-of-care ultrasound in the ED evaluation of patients presenting with chest pain and shortness of breath. Am J Emerg Med.

[33] Macaskill P, Takwoingi Y, Deeks JJ, C. G. Chapter 9: Understanding meta-analysis. Draft version (2022) for inclusion. London: Cochrane. In: Cochrane Handbook for Systematic Reviews of Diagnostic Test Accuracy Version 2.

[34] Deeks JJBP, Leeflang MM, Takwoingi Y. Cochrane Handbook for Systematic Reviews of Diagnostic Test Accuracy Version 2: London: Cochrane; 2022.

[35] Salameh JP, Bossuyt PM, McGrath TA, Thombs BD, Hyde CJ, Macaskill P (2020). Preferred reporting items for systematic review and meta-analysis of diagnostic test accuracy studies (PRISMA-DTA): explanation, elaboration, and checklist. BMJ.

[36] Spry C, Mierzwinski-Urban M (2018). The impact of the peer review of literature search strategies in support of rapid review reports. Res Synth Methods.

[37] Kesmodel US (2018). Information bias in epidemiological studies with a special focus on obstetrics and gynecology. Acta Obstet Gynecol Scand.

[38] Altman DG, Bland JM (2007). Missing data. BMJ.

[39] Eisen A, Giugliano RP, Braunwald E (2016). Updates on acute coronary syndrome: a review. JAMA Cardiol.

[40] Byrne RA, Rossello X, Coughlan JJ, Barbato E, Berry C, Chieffo A (2023). 2023 ESC guidelines for the management of acute coronary syndromes. Eur Heart J.

[41] Tramer MR, Reynolds DJ, Moore RA, McQuay HJ (1997). Impact of covert duplicate publication on meta-analysis: a case study. BMJ.

[42] von Elm E, Poglia G, Walder B, Tramer MR (2004). Different patterns of duplicate publication: an analysis of articles used in systematic reviews. JAMA.

[43] Whiting PF, Rutjes AW, Westwood ME, Mallett S, Deeks JJ, Reitsma JB (2011). QUADAS-2: a revised tool for the quality assessment of diagnostic accuracy studies. Ann Intern Med.

[44] Reitsma JBRA, Whiting P, Yang B, Leeflang MM, Bossuyt PM, Deeks JJ. Chapter 8: Assessing risk of bias and applicability. Cochrane, 2023. In: Cochrane handbook for systematic reviews of diagnostic test accuracy version 20 (updated July 2023). Available from: Available from https://training.cochrane.org/handbook-diagnostic-test-accuracy/current.

[45] Reitsma JB, Glas AS, Rutjes AW, Scholten RJ, Bossuyt PM, Zwinderman AH (2005). Bivariate analysis of sensitivity and specificity produces informative summary measures in diagnostic reviews. J Clin Epidemiol.

[46] Harbord RM, Whiting P, Sterne JA, Egger M, Deeks JJ, Shang A, Bachmann LM (2008). An empirical comparison of methods for meta-analysis of diagnostic accuracy showed hierarchical models are necessary. J Clin Epidemiol.

[47] Macaskill PTY, Deeks JJ, Gatsonis C. Chapter 9: Understanding meta-analysis. Draft version (2022) for inclusion. In: Cochrane Handbook for Systematic Reviews of Diagnostic Test Accuracy Version 2 [Internet]. London: Cochrane.

[48] Richardson M, Garner P, Donegan S (2019). Interpretation of subgroup analyses in systematic reviews: a tutorial. Clin Epidemiol Global Health.

[49] Nyaga VN, Arbyn M (2022). Metadta: a Stata command for meta-analysis and meta-regression of diagnostic test accuracy data—a tutorial. Arch Public Health.

[50] Takwoingi YDN, Schiller I, Rücker G, Jones HE, Partlett C, Macaskill P. Chapter 10: Undertaking meta-analysis. Cochrane 2023. In: Cochrane Handbook for Systematic Reviews of Diagnostic Test Accuracy Version 20 (updated July 2023) [Internet]. Available from: Available from: https://training.cochrane.org/handbook-diagnostic-test-accuracy/current.

[51] Nyaga VN, Arbyn M (2023). Comparison and validation of metadta for meta-analysis of diagnostic test accuracy studies. Res Synth Methods.

[52] Deeks JJ, Macaskill P, Irwig L (2005). The performance of tests of publication bias and other sample size effects in systematic reviews of diagnostic test accuracy was assessed. J Clin Epidemiol.

[53] Schunemann HJ, Mustafa RA, Brozek J, Steingart KR, Leeflang M, Murad MH (2020). GRADE guidelines: 21 part 2. Test accuracy: inconsistency, imprecision, publication bias, and other domains for rating the certainty of evidence and presenting it in evidence profiles and summary of findings tables. J Clin Epidemiol.

[54] Schunemann HJ, Mustafa RA, Brozek J, Steingart KR, Leeflang M, Murad MH (2020). GRADE guidelines: 21 part 1. Study design, risk of bias, and indirectness in rating the certainty across a body of evidence for test accuracy. J Clin Epidemiol.

[55] GRADEpro GDT: GRADEpro Guideline Development Tool [Software]. McMaster University and Evidence Prime; 2022.

[56] Allen MOJK, Farkouh ME, Zinsmeister AR, Smars PA, Seward JB, Reeder GS. The rate of emergency room echocardiography in imaging patients with chest pain at intermediate risk for acute myocardial infarction: a substudy of CHEER. J Am College Cardiol 1998.

[57] Çevrim Ö, Boydak B, Yürüktümen A, Kiyan GS, Ersel M, Uz İ (2023). The diagnostic value of echocardiography performed by an emergency medicine physician in the diagnosis of acute coronary syndrome: a comparative study with cardiologist. J Diagnos Med Sonography.

[58] Dahlslett T, Karlsen S, Grenne B, Eek C, Sjoli B, Skulstad H (2014). Early assessment of strain echocardiography can accurately exclude significant coronary artery stenosis in suspected non-ST-segment elevation acute coronary syndrome. J Am Soc Echocardiogr.

[59] De Loizaga SLT, Dagan A, Pacheco F, Thomas D, Oligino E, Herbst M (2014). Focused echocardiograms by emergency physicians for the risk stratification of patients with chest pain. Academic Emerg Med.

[60] Di Pasquale PCS, Pipitone F, Marenghini G, Sarullo FM, Cacia A, Paterna S (2005). Utility of the immediate 2-dimensional echocardiography and troponin T test combination for diagnosing non-ST elevation acute coronary syndromes in patients with T-wave negative and non-diagnostic electrocardiogram. J Clin Basic Cardiol.

[61] Hickman M, Swinburn JM, Senior R (2004). Wall thickening assessment with tissue harmonic echocardiography results in improved risk stratification for patients with non-ST-segment elevation acute chest pain. Eur J Echocardiogr.

[62] Kang DH, Kang SJ, Song JM, Choi KJ, Hong MK, Song JK (2005). Efficacy of myocardial contrast echocardiography in the diagnosis and risk stratification of acute coronary syndrome. Am J Cardiol.

[63] Kontos MC, Arrowood JA, Jesse RL, Ornato JP, Paulsen WH, Tatum JL, Nixon JV (1998). Comparison between 2-dimensional echocardiography and myocardial perfusion imaging in the emergency department in patients with possible myocardial ischemia. Am Heart J.

[64] Korosoglou G, Labadze N, Hansen A, Selter C, Giannitsis E, Katus H, Kuecherer H (2004). Usefulness of real-time myocardial perfusion imaging in the evaluation of patients with first time chest pain. Am J Cardiol.

[65] Kountana E, Tziomalos K, Semertzidis P, Dogrammatzi F, Slavakis A, Douma S (2013). Comparison of the diagnostic accuracy of ischemia-modified albumin and echocardiography in patients with acute chest pain. Exp Clin Cardiol.

[66] Lee M, Chang SA, Cho EJ, Park SJ, Choi JO, Lee SC (2015). Role of strain values using automated function imaging on transthoracic echocardiography for the assessment of acute chest pain in emergency department. Int J Cardiovasc Imaging.

[67] Oh JKSC, Miller FA (1985). Role of two-dimensional echocardiography in the emergency room. Echocardiography.

[68] Onishi TWT, Fujita M, Mizukami Y, Sakata Y, Nakatani S, Nanto S, Uematsu M. Risk stratification of chest pain in emergency department using non-provocative echocardiography combined with tissue doppler dyssynchrony imaging. Eur Heart J Cardiovasc Imaging. 2013;2:ii106.

[69] Peels CH, Visser CA, Kupper AJ, Visser FC, Roos JP (1990). Usefulness of two-dimensional echocardiography for immediate detection of myocardial ischemia in the emergency room. Am J Cardiol.

[70] Sabia P, Afrookteh A, Touchstone DA, Keller MW, Esquivel L, Kaul S (1991). Value of regional wall motion abnormality in the emergency room diagnosis of acute myocardial infarction. A prospective study using two-dimensional echocardiography. Circulation.

[71] Shiran A, Blondheim DS, Shimoni S, Jabarren M, Rosenmann D, Sagie A (2017). Two-dimensional strain echocardiography for diagnosing chest pain in the emergency room: a multicentre prospective study by the Israeli echo research group. Eur Heart J Cardiovasc Imaging.

[72] Sobczyk D, Nycz K, Andruszkiewicz P (2015). Validity of a 5-minute focused echocardiography with A-F mnemonic performed by non-echocardiographers in the management of patients with acute chest pain. Cardiovasc Ultrasound.

[73] Swinburn JM, Stubbs P, Soman P, Collinson P, Lahiri A, Senior R (2002). Independent value of tissue harmonic echocardiography for risk stratification in patients with non-ST-segment elevation acute chest pain. J Am Soc Echocardiogr.

[74] Weston P, Alexander JH, Patel MR, Maynard C, Crawford L, Wagner GS (2004). Hand-held echocardiographic examination of patients with symptoms of acute coronary syndromes in the emergency department: the 30-day outcome associated with normal left ventricular wall motion. Am Heart J.

[75] Wilben V, Limbad D, Bs B, Ts S, Kanchi M (2021). Recommendation for inclusion of surface echocardiography in evaluation of chest pain in acute emergency care. J Cardiac Critical Care TSS.

[76] Atar S, Feldman A, Darawshe A, Siegel RJ, Rosenfeld T (2004). Utility and diagnostic accuracy of hand-carried ultrasound for emergency room evaluation of chest pain. Am J Cardiol.

[77] Chandra ABU, Kurkowski E, Menlove S, Vermeulen M, Carmody K (2015). 356 Emergency physician-performed echocardiography as a predictor of cardiac events in patients presenting with symptoms of acute coronary syndrome. Annals Emerg Med.

[78] Cohen JF, Korevaar DA, Wang J, Leeflang MM, Bossuyt PM (2016). Meta-epidemiologic study showed frequent time trends in summary estimates from meta-analyses of diagnostic accuracy studies. J Clin Epidemiol.

[79] Thygesen K, Alpert JS, Jaffe AS, Chaitman BR, Bax JJ, Morrow DA (2018). Fourth universal definition of myocardial infarction (2018). J Am Coll Cardiol.

[80] Kaier TE, Alaour B, Marber M (2021). Cardiac troponin and defining myocardial infarction. Cardiovasc Res.

[81] Collet JP, Thiele H, Barbato E, Barthelemy O, Bauersachs J, Bhatt DL (2021). 2020 ESC Guidelines for the management of acute coronary syndromes in patients presenting without persistent ST-segment elevation. Eur Heart J.

[82] Body R, Carley S, Wibberley C, McDowell G, Ferguson J, Mackway-Jones K (2010). The value of symptoms and signs in the emergent diagnosis of acute coronary syndromes. Resuscitation.

[83] Dezman ZD, Mattu A, Body R (2017). Utility of the history and physical examination in the detection of acute coronary syndromes in emergency department patients. West J Emerg Med.

[84] Herring N, Paterson DJ (2006). ECG diagnosis of acute ischaemia and infarction: past, present and future. QJM.

[85] Ghadrdoost B, Haghjoo M, Firouzi A (2015). Accuracy of cardiogoniometry compared with electrocardiography in the diagnosis of coronary artery disease. Res Cardiovasc Med.

[86] Larson DM, Menssen KM, Sharkey SW, Duval S, Schwartz RS, Harris J (2007). "False-positive" cardiac catheterization laboratory activation among patients with suspected ST-segment elevation myocardial infarction. JAMA.

[87] National Institute for Health and Care Excellence. High-sensitivity troponin tests for the early rule out of NSTEMI. NICE Diagnostics guidance (DG40). 2020.

[88] Novis DA, Jones BA, Dale JC, Walsh MK, College of American P. Biochemical markers of myocardial injury test turnaround time: a College of American Pathologists Q-Probes study of 7020 troponin and 4368 creatine kinase-MB determinations in 159 institutions. Arch Pathol Lab Med. 2004;128(2):158–64. 10.5858/2004-128-158-BMOMIT 14736289

[89] Lazar DR, Lazar FL, Homorodean C, Cainap C, Focsan M, Cainap S, Olinic DM (2022). High-sensitivity troponin: a review on characteristics, assessment, and clinical implications. Dis Markers.

[90] Mancuso FJ, Siqueira VN, Moises VA, Gois AF, Paola AA, Carvalho AC, Campos O (2014). Focused cardiac ultrasound using a pocket-size device in the emergency room. Arq Bras Cardiol.

[91] Sobczyk D, Nycz K, Zmudka K (2014). Usefulness of limited echocardiography with A-F mnemonic in patients with suspected nonST-segment elevation acute coronary syndrome. Pol Arch Med Wewn.

[92] Guner NG, Yurumez Y, Yucel M, Alacam M, Guner ST, Ercan B (2020). Effects of point-of-care ultrasonography on the diagnostic process of patients admitted to the emergency department with chest pain: a randomised controlled trial. J Coll Physicians Surg Pak.

[93] Kobal SL, Trento L, Baharami S, Tolstrup K, Naqvi TZ, Cercek B (2005). Comparison of effectiveness of hand-carried ultrasound to bedside cardiovascular physical examination. Am J Cardiol.

[94] Dodd S, Clarke M, Becker L, Mavergames C, Fish R, Williamson PR (2018). A taxonomy has been developed for outcomes in medical research to help improve knowledge discovery. J Clin Epidemiol.

