# Supplementary material for: The diagnostic accuracy of cardiac ultrasound for acute myocardial ischemia in the emergency department: a systematic review and meta-analysis
Source: Scand J Trauma Resusc Emerg Med. 2024 Mar 11;32:19. doi: 10.1186/s13049-024-01192-3 (PMC10926567; doi:10.1186/s13049-024-01192-3)
Supplement: Supplementary file 1 — Additional file 1. e-Appendix 2: Search Strategy. [file 13049_2024_1192_MOESM1_ESM.docx]

electronic supplementary material

Tabla de contenido

[e- Appendix 1. PRISMA-DTA Checklist 3](#_Toc160029423)

[e-Appendix 2. Search Strategy 4](#_Toc160029424)

[e-Appendix 3. Data Extraction Form 8](#_Toc160029425)

[e-Appendix 4. Additional characteristics of included studies 13](#_Toc160029426)

[e-Appendix 5. Quality assessment of included studies 16](#_Toc160029427)

[Patient Selection 16](#_Toc160029428)

[Index Test 16](#_Toc160029429)

[Reference Standard 17](#_Toc160029430)

[Flow and Timing 18](#_Toc160029431)

[e-Appendix 6. GRADE assessment of certainty of evidence 19](#_Toc160029432)

[e-Appendix 7. Subgroup analyses 21](#_Toc160029433)

[Subgroup analysis by patient spectrum 21](#_Toc160029434)

[Subgroup analysis by ultrasound operator 23](#_Toc160029435)

[Subgroup analysis by timing of ultrasound 24](#_Toc160029436)

[Subgroup analysis by type of ultrasound device 26](#_Toc160029437)

[Subgroup analysis by ultrasound scanning protocol used. 27](#_Toc160029438)

[Subgroup analysis by reference standard 29](#_Toc160029439)

[Subgroup analysis by target condition 31](#_Toc160029440)

[e-Appendix 8. Sensitivity Analyses 33](#_Toc160029441)

[Removing studies with high risk of bias 33](#_Toc160029442)

[Removing studies with inappropriate reference standard 34](#_Toc160029443)

[Removing unpublished studies (conference abstracts). 35](#_Toc160029444)

[e-Appendix 9. Publication Bias 37](#_Toc160029445)

[e-Appendix 10. Deviations from the original protocol 38](#_Toc160029446)

[REFERENCES 38](#_Toc160029447)

# e- Appendix 1. PRISMA-DTA Checklist

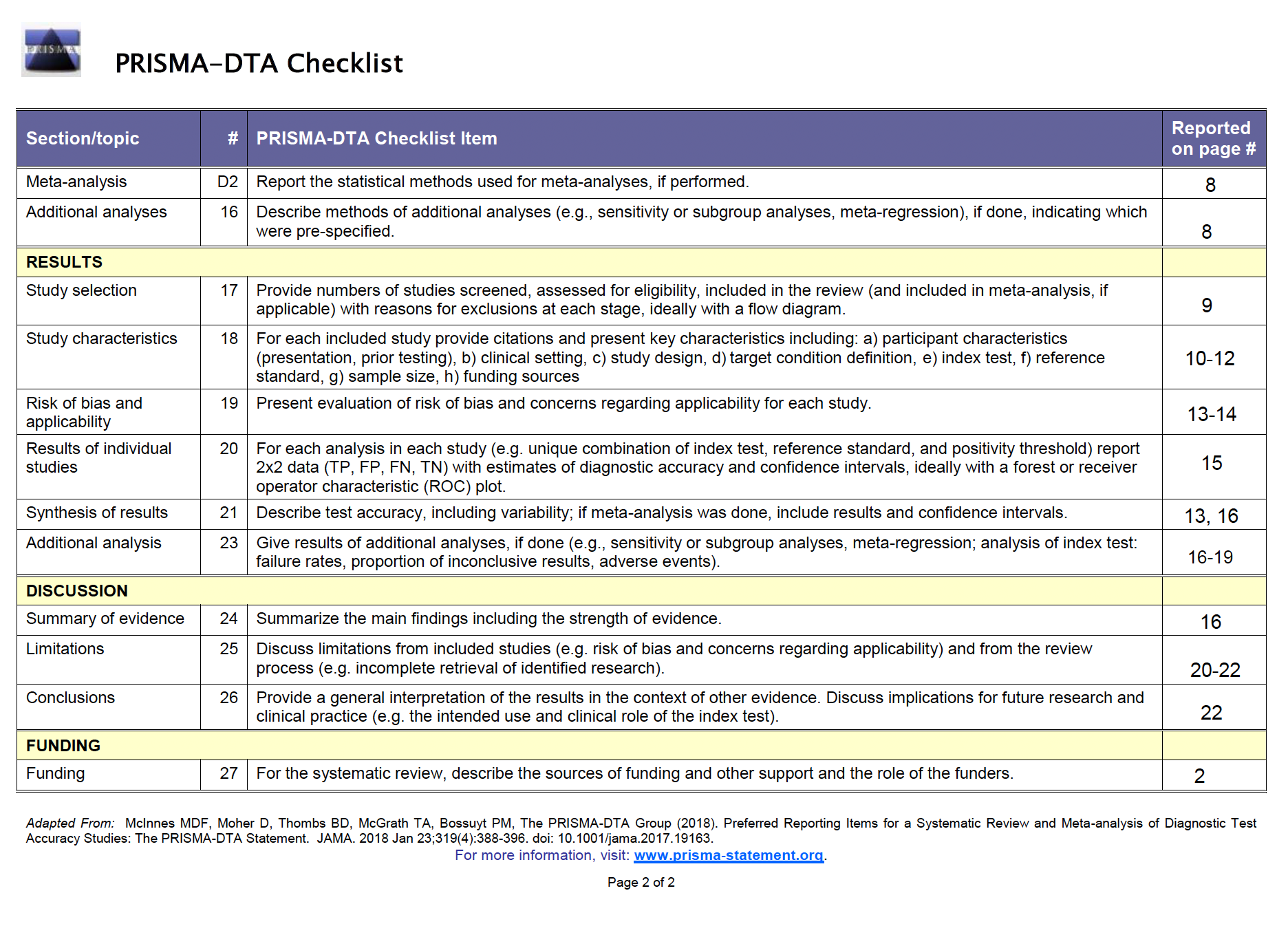


# e-Appendix 2. Search Strategy

# e-Appendix 3. Data Extraction Form

Data extraction was conducted in Covidence® which is a web-based collaboration software platform that streamlines the production of systematic and other literature reviews. Covidence systematic review software, Veritas Health Innovation, Melbourne, Australia. Available at [www.covidence.org](http://www.covidence.org).

The following data extraction form was created inside covidence platform:

**General information**

**Study ID**

**Title**

**Lead Author (Last name, Name)**

**Lead author contact details**

**Country in which the study was conducted**

1. United States
2. UK
3. Australia
4. Italy
5. Turkey
6. Other

**City**

**Number of Centers**

1. Single Center
2. Two centers
3. Three centers
4. Other

**Main Research Hospital**

**Language**

1. English
2. Spanish
3. Portuguese
4. German
5. Other

**Characteristics of included studies**

**Methods**

**Aim of study**

**Study design**

1. Randomized controlled trial
2. Non-randomized experimental study
3. Cohort study
4. Cross sectional study
5. Case control study
6. Prevalence study
7. Diagnostic test accuracy study
8. Clinical prediction rule
9. Other

**Start date (Recruitment) dd/mm/aa**

**End date (Recruitment) dd/mm/aa**

**Study funding sources**

1. Industry
2. Government
3. Institutional
4. Non-Profit Organization
5. None
6. Not reported/Unclear
7. Other

**Conflict of Interest**

1. None
2. Not reported/Unclear
3. Other

**Participants**

**Inclusion criteria**

**Exclusion criteria**

**Method of recruitment of participants**

1. Consecutive
2. Convenience
3. Random
4. Not Reported/ Unclear
5. Other

**Ultrasound**

**Machine**

1. Hand-Held
2. POCUS Cart/Tray device
3. Full Echocardiography Machine
4. Full portable Ultrasound Machine
5. Not Reported/Unclear
6. Other

**Ultrasound Protocol**

1. Full Transthoracic Echocardiography (TTE)
2. Focused Cardiac Ultrasound or Cardiac POCUS
3. Limited TTE
4. POCUS+ (Cardiac AND other organs, i.e.: lung)
5. Not Reported/Unclear
6. Other

**Ultrasound Operator**

1. Emergency physician
2. Emergency medicine resident
3. Cardiologist
4. Cardiology fellow
5. Sonographer/ultrasound technician
6. Unclear /Not reported
7. Other

**Operator Training/Experience**

**Ultrasound Interpretation**

1. Interpreted at bedside
2. Interpreted in cardiology lab / videos
3. Unclear/Not reported
4. Other

**Ultrasound Time limit**

1. At admission or short after admission
2. < 2 hours
3. < 6 hours
4. <12 hours
5. < 24 hours
6. Not reported/Unclear
7. Other

**Positive Findings for Ischemia: Index Test positive definition**

1. RWMA only
2. RWMA + LVEF reduction
3. Not Reported/Unclear
4. Other

**Timing of Reference Standard**

**Reference Standard**

Check all that apply

1. Chart Review by Independent person
2. Final diagnosis by treating clinicians
3. Coronary Angiography
4. Echocardiography by cardiology
5. EKG changes and Cardiac Enzymes
6. Stress- induced ischemia testing
7. Non-invasive Testing
8. Not reported
9. Other

**Outcomes**

**Total number of included patients**

**Baseline Population Characteristics**

|  | **Mean** | **SD** | **Median** | **IQR** | **Range (min-max)** |
| --- | --- | --- | --- | --- | --- |
| **Age** |  |  |  |  |  |

**Admission EKG**

Admission EKG findings

1. Normal EKG
2. Non-Diagnostic EKG
3. T wave inversion or ST depression
4. LBBB
5. All EKGs (Including STEMI)
6. All EKGs (Excluding STEMI)
7. Not reported/unclear
8. Other

**Troponin**

1. Negative initial troponin
2. Negative serial troponin
3. All patients with chest pain, independent of troponin result
4. Not reported/Unclear
5. Other

**Male (%)**

**History of Cardiac Disease (%)**

**Inadequate US Window (%)**

**Time from Admission to US**

|  | **Mean (SD)** | **Median (IQR)** | **Range (min-max)** |
| --- | --- | --- | --- |
| **Time** |  |  |  |

**Data 2x2 Diagnostic Accuracy**

|  | **Ischemia (+)** | **Ischemia (-)** | **Total** |
| --- | --- | --- | --- |
| **Ultrasound (+)** |  |  |  |
| **Ultrasound (-)** |  |  |  |
| **Total** |  |  |  |

**Results**

|  | **Result** |
| --- | --- |
| **Sensitivity (%)** |  |
| **Specificity (%)** |  |
| **PPV (%)** |  |
| **NPV (%)** |  |
| **LR +** |  |
| **LR -** |  |
| **Prevalence (%)** |  |
| **AUC** |  |

**Differential Diagnosis Detected (%)**

**Notes**

# e-Appendix 4. Additional characteristics of included studies

# e-Appendix 5. Quality assessment of included studies

## Patient Selection

There was high or unclear risk of bias in 26 studies (90%). Consecutive sampling was described only in 11 studies,^1-11^ with convenience sampling in 5 studies^12-16^ and unclear sampling in 13 studies.^17-29^ A non-consecutive sampling method is associated with an overestimation of diagnostic accuracy.^30^ A case control design was avoided in all studies. However, the inappropriate exclusion of patients with previous heart disease (coronary disease, heart failure, valvopathies, etc.), patients with low or high risk of acute coronary syndrome and/or patients with inadequate ultrasound images that was found in many studies, can overestimate diagnostic accuracy results by not including “difficult-to-diagnose” patients, straying off the spectrum of patients with chest pain encountered in clinical practice, and therefore were judged to be at high risk of bias.^30-32^ Similarly, these exclusions limit the applicability of the study results to the target population of patients admitted with chest pain in the ED, which commonly includes patients with previous heart failure and/or coronary artery disease, and also may have some inadequate echocardiographic windows.

## Index Test

The cardiac ultrasound was conducted early in the clinical pathway of the patients with chest pain in the ED, and it was interpreted without the knowledge of reference standard, which occurred later in the hospital stay in all studies. A definition for index test positivity was reported in all studies except for one which was a conference abstract.^29^ Twenty-three studies used RWMA as definition of a positive index test,^1,3,4,6,7,9-12,14,15,17,19,20,22-29^ and six studies used a combination of RWMA and/or reduced left ventricular ejection fraction (LEVF).^2,5,8,13,16,18^ Variations in technology with the introduction of THI in late 1990s and the unclear the effect that this had in diagnostic accuracy and the lack of a description of ultrasound operators and their previous training resulted in unclear concerns of applicability in most studies (17). High concerns of applicability were found in two studies.^15,16^ In one study, cardiac ultrasound was performed by 3^rd^ year medical students with very limited training, which was considered to have very limited generalizability,^16^ and the other study compared admission cardiac ultrasounds with previous patient’s echocardiographic studies as part of the study protocol, which although this would be ideal, the possibility of accomplishing this in real life emergency settings for all patients is very low.^15^ The remaining studies were judged to have low concerns of applicability (11 studies).^1,2,4,6,10-13,19,22^

## Reference Standard

The reference standard domain was judged to have a high risk of bias in 16 studies due to lack of blinding to index test results or the reference standard was not likely to correctly classify myocardial ischemia (i.e., cardiac enzymes only).^3,4,8-10,13,15,16,20,22-24,26,28,29^ Eleven studies had an unclear risk of bias because patients received different tests and investigations as part of the reference standard diagnostic pathway, and it was not clear what these tests were and how they were decided upon.^1,2,5,7,11,12,14,17,18,21,25,27^ Finally, only two studies were judged to be as low risk of bias.^6,19^ There were high concerns of applicability in 13 studies due to the use of CK, CK-MB and LDH as cardiac biomarkers, which are not currently used in the diagnostic pathway of myocardial ischemia and in some studies, the definition of myocardial ischemia as target condition does not match the current definition.^3,4,7-9,12,13,15,16,20,23,27,29^ In 11 studies, the concerns of applicability were unclear, because the reference standard or the target condition were not clearly defined.^2,5,10,11,14,17,18,21,24-26,28^ Four studies were judged to have low concerns of applicability.^1,6,19,22^

## Flow and Timing

Regarding flow and timing, 13 studies were judged to be at high risk of bias. In 11 of these 13 studies, high risk of bias was due to partial or differential verification or the exclusion of patients from the analysis (i.e., patients with inadequate echocardiographic windows).^4,8,9,12,15,16,22,24,26-28^ One study had an inappropriate interval of 3 weeks for the conduct of the reference standard^25^ and in another study, the echocardiographic study was part of the final diagnosis,^10^ thus incurring incorporation bias. In eight studies, the risk of bias was unclear because patients received different tests and investigations as part of the reference standard diagnostic pathway and it is not clear what these tests were and how they were decided upon, and finally, 8 studies were at low risk of bias.

# e-Appendix 6. GRADE assessment of certainty of evidence

Based on GRADE, the overall assessment of certainty was very low.^33-35^ Risk of bias was rated “very serious” because 90% of studies had a high risk of bias in one or more domain, mainly deriving from patient selection bias due to exclusion of patients with previous heart disease, patients with low or high-risk of myocardial ischemia and/or difficult ultrasound windows, and from bias in the reference standard that was unblinded and/or did not correctly classify myocardial ischemia according to current clinical practice. Indirectness was rated “very serious” because 90% of studies had high concerns of applicability in one or more domain, primarily patient selection, and reference standard. Inconsistency was rated “not serious” as substantial heterogeneity was explained by differences in patient spectrum, timing of ultrasound, target condition and reference standard. Unexplained heterogeneity was not considered serious. Imprecision was rated “serious” for sensitivity because the confidence interval was wide, and the lower limit of the interval is below 70% with important clinical consequences for false negative cases. Imprecision for specificity was rated “not serious” since the lower limit of the confidence interval was above 80%.

# e-Appendix 7. Subgroup analyses

## Subgroup analysis by patient spectrum

Studies differed in the spectrum of patients included. Some studies included patients independent of their past medical history, while other studies excluded patients if previous heart disease (coronary heart disease, heart failure or valvopathy) was identified. Thirteen studies excluded patients with previous heart disease^3-7,11,14,19,22,23,25,27,28^ while another 13 studies included patients independent of their past medical history on heart disease.^1,2,8-10,13,15-18,20,21,29^ Three studies did not have information on the inclusion or exclusion of patients according to previous heart disease.^12,24,26^ The paired Forest plot of sensitivity and specificity is shown in e-Figure 1. There was a higher specificity in studies that excluded patients with previous heart disease (91.0%, 95%CI 84.1 to 95.1%) compared to studies that included patients with or without heart disease (78.0%, 95%CI 66.1 to 86.6%, *P* =0.029), and with a less degree of uncertainty, as depicted in the SROC plot in e-Figure 2. There was substantial heterogeneity in specificity between studies in the group that included patients with heart disease, thus, the validity of the significant effect remains uncertain. Sensitivity was similar across both groups. The higher specificity in patients without heart disease has a clinical and physiological rationale since excluding patients with previous regional wall motion abnormalities (RWMAs) or reduced left ventricular ejection fraction from chronic heart disease, allows an easier identification of new RWMAs and thus decrease the number of false positives. There was substantial heterogeneity between studies in both groups, as displayed by the wide prediction region.


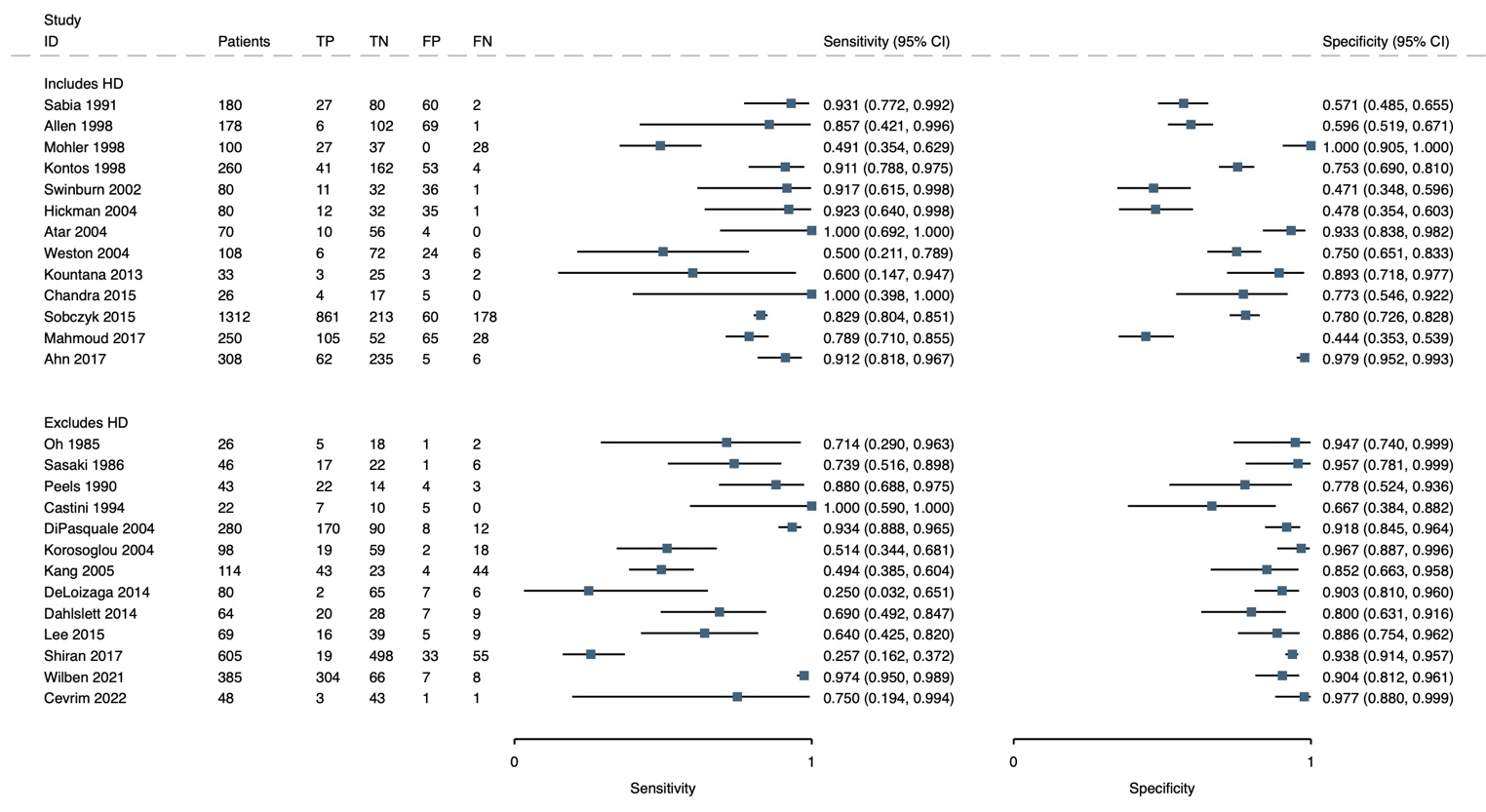
e-Figure 1. Paired forest plot of sensitivity and specificity by patient spectrum. HD Heart Disease, TP= true positive, TN = true negative, FP= false positive, FN= false negative.


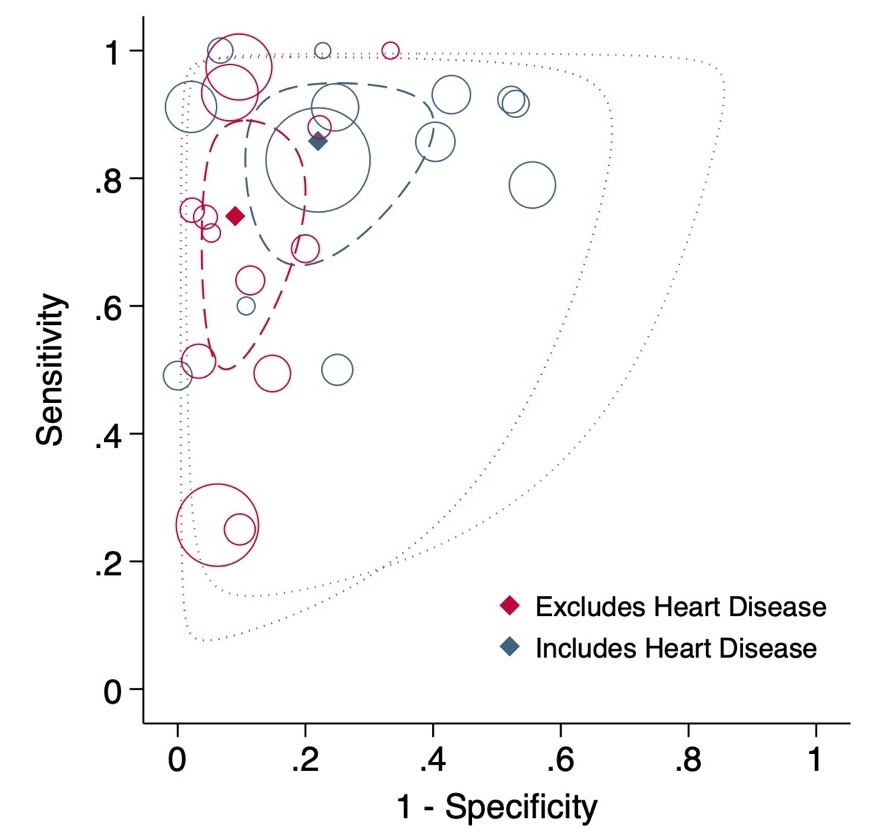


e-Figure 2. SROC plot of sensitivity and specificity by patient spectrum. Each study is represented by a circle, with the size of the circle indicating the size of the sample.

## Subgroup analysis by ultrasound operator

In six studies the ultrasound operators were from the cardiology department (cardiologists, cardiology fellows or sonographers), in another six studies the operators were from the emergency department (emergency physicians or residents), and in three studies the operators were experienced sonographers with no clear description of training background or department of origin. The paired Forest plot of sensitivity and specificity is depicted in e-Figure 3. There was no information on the ultrasound operator in 14 studies. There was no significant difference in sensitivity (*P* = 0.72) and specificity (*P* = 0.983) across groups. Overall, there was greater uncertainty and heterogeneity in estimates from studies from emergency medicine and sonographers (e-Figure 4).


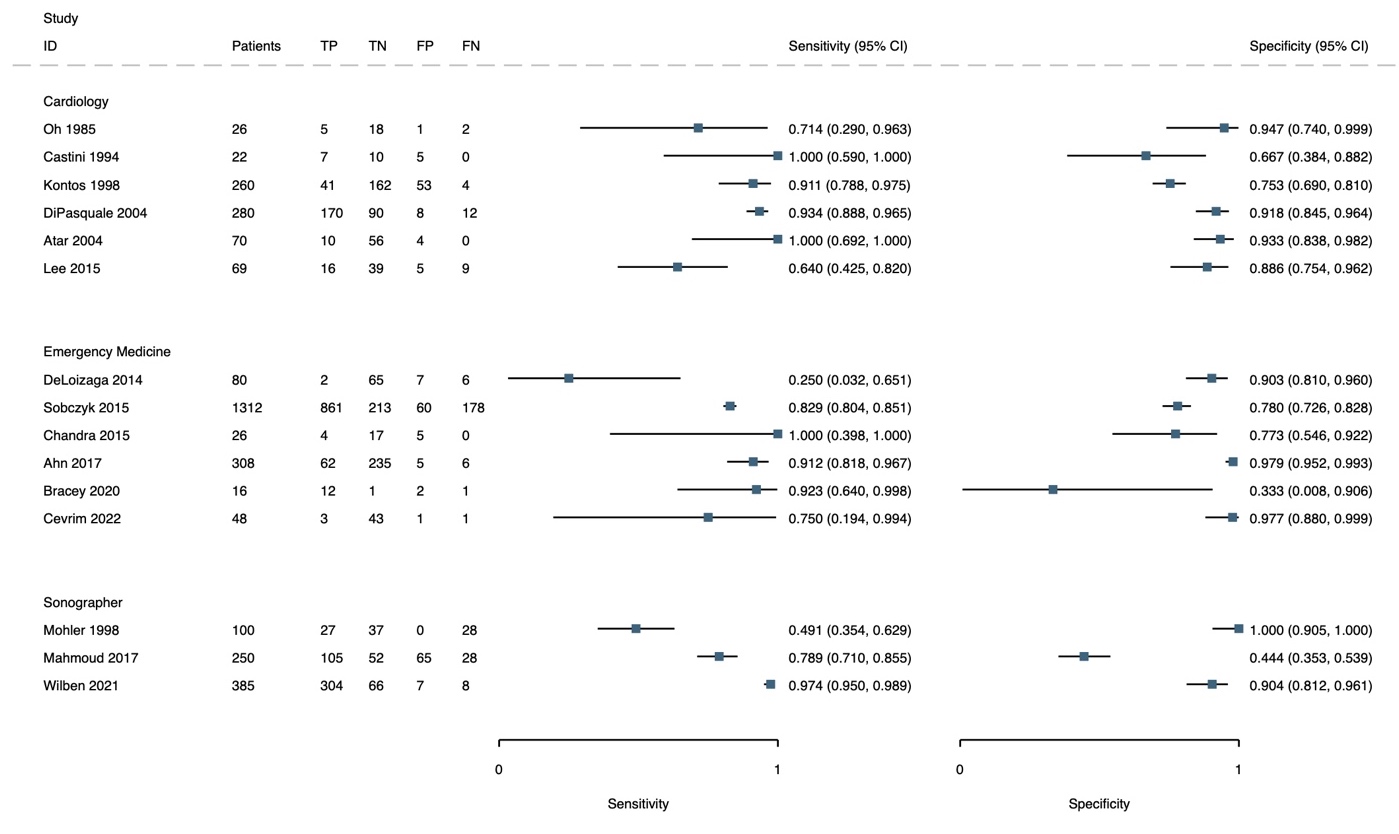


e-Figure 3. Paired Forest plot of sensitivity and specificity by operator. TP= true positive, TN = true negative, FP= false positive, FN= false negative.


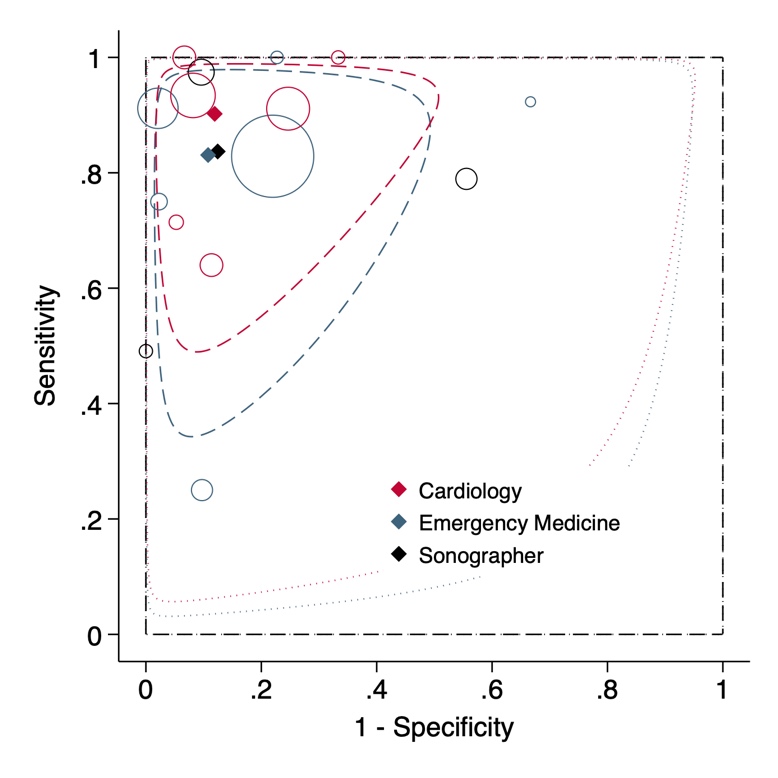


e-Figure 4. SROC plot of sensitivity and specificity by operator. Each study is represented by a circle, with the size of the circle indicating the size of the sample.

## Subgroup analysis by timing of ultrasound

Timing of ultrasound differed across studies. Nine studies conducted the ultrasound at admission to the ED, while four studies reported different times during the first 24 hours of ED admission. Paired Forest plot is shown in e-Figure 5. There was a significantly higher sensitivity in studies where ultrasound was conducted at admission or immediately after admission (89.5%, 95%CI 78.9 to 95.1), compared to studies where the timing of ultrasound was conducted at any time during the first 24 hours (62.8%, 95%CI 34.6 to 84.3); with a *P* value of 0.038 (e-Figure 6). There is a clinical and pathophysiological rationale for this difference in sensitivity since RWMA are present during ongoing myocardial ischemia and are transient, lasting from minutes to a few hours after an ischemic episode.^6,8,36,37^ When the cardiac ultrasound is performed at ED admission, there is a higher probability that patients still have ongoing pain, or the pain has subsided a short time ago. This increases the probability of that RWMA are still present and detectable by ultrasound. Specificity was similar across both groups. There was greater uncertainty in estimates from studies where ultrasound was performed at any time during the first 24 hours as depicted in the large 95% confidence region of the SROC plot (e-Figure 6) and there was substantial heterogeneity between studies in both groups represented in the wide 95% prediction region.


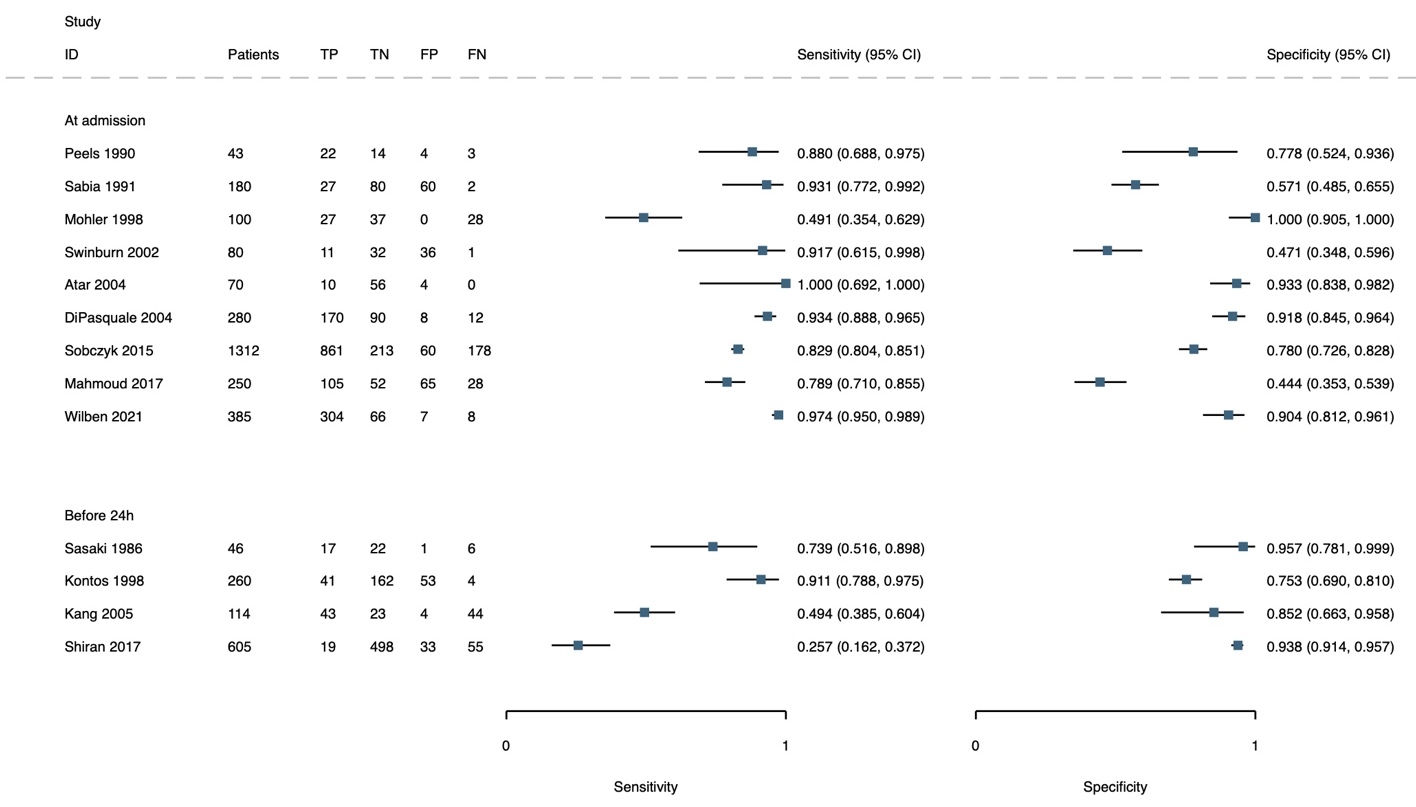


e-Figure 5. Paired Forest plot of sensitivity and specificity by timing of ultrasound. TP= true positive, TN = true negative, FP= false positive, FN= false negative.


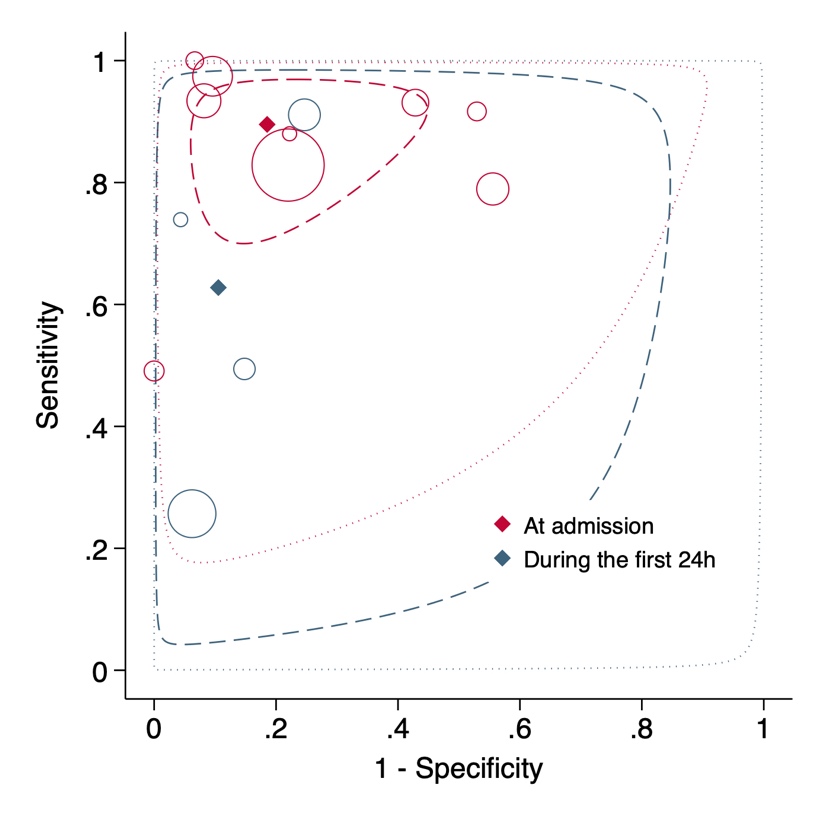


e-Figure 6. SROC plot of sensitivity and specificity by timing of ultrasound. Each study is represented by a circle, with the size of the circle indicating the size of the sample.

## Subgroup analysis by type of ultrasound device

Different devices were used to conduct the cardiac ultrasound test across studies. In 20 studies, operators used full echocardiography devices and in 4 studies POCUS cart/tray-based or handheld devices were used (e-Figure 7). In five studies, device used was not reported. Sensitivity and specificity were similar across groups, with much greater uncertainty in studies using Cart/tray or handheld devices compared to echocardiography devices as depicted by the large 95% confidence region compared to the more condensed region in studies using echocardiography devices. There was substantial heterogeneity between studies in both groups represented in the large 95% prediction region (e-Figure 8).


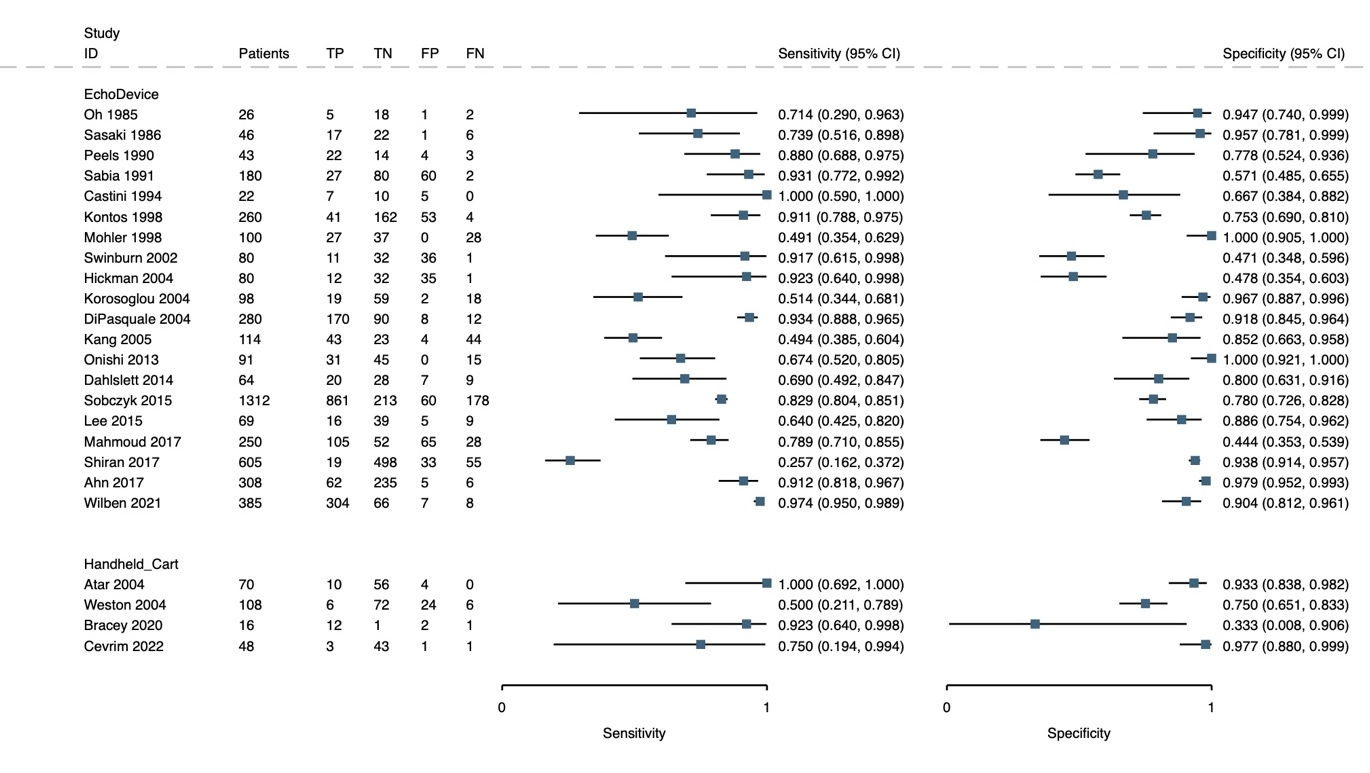


e-Figure 7. Paired Forest plot of sensitivity and specificity by type of ultrasound device. TP= true positive, TN = true negative, FP= false positive, FN= false negative.


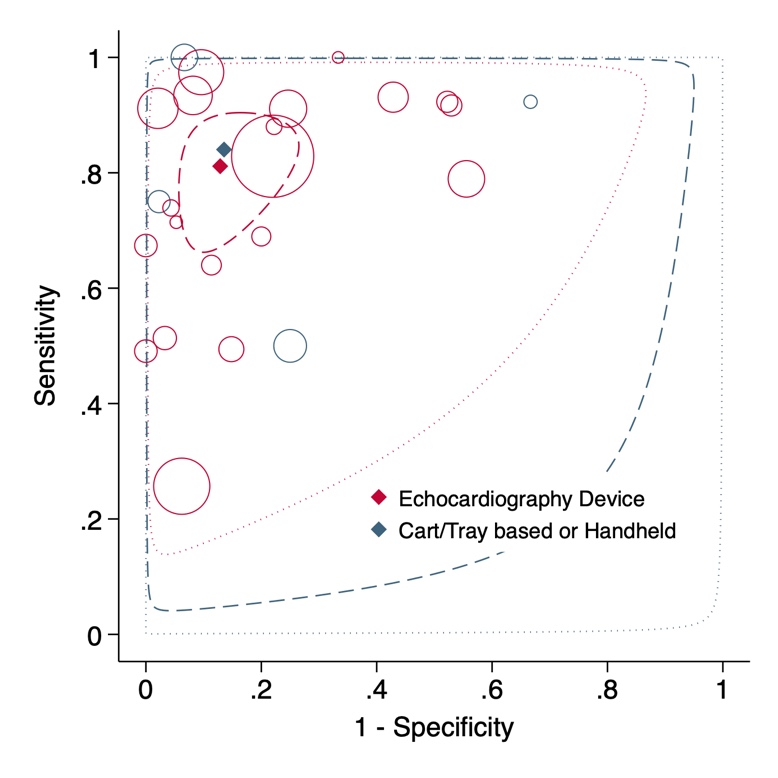


e-Figure 8. SROC plot of sensitivity and specificity by type of ultrasound device. Each study is represented by a circle, with the size of the circle indicating the size of the sample.

## Subgroup analysis by ultrasound scanning protocol used.

Cardiac ultrasound protocol used to assess the presence of RWMA was different across studies. Some studies used a POCUS protocol, that uses specific 4 to 5 cardiac views and other studies used a limited-TTE or a complete echocardiography protocol (TTE). Limited TTE and TTE were grouped together since limited TTE requires similar training and technique and uses the same cardiac windows for the assessment of RWMA as a complete echocardiographic exam. Sensitivity and specificity estimates were similar for studies that used a POCUS protocol compared to studies that used Limited-TTE/TTE (e-Figure 9). There was considerable uncertainty in estimates from POCUS studies and, although between-study heterogeneity was substantial in both groups, it was also greater in POCUS group, as it can be depicted in the 95% confidence and 95% prediction regions (e-Figure 10).


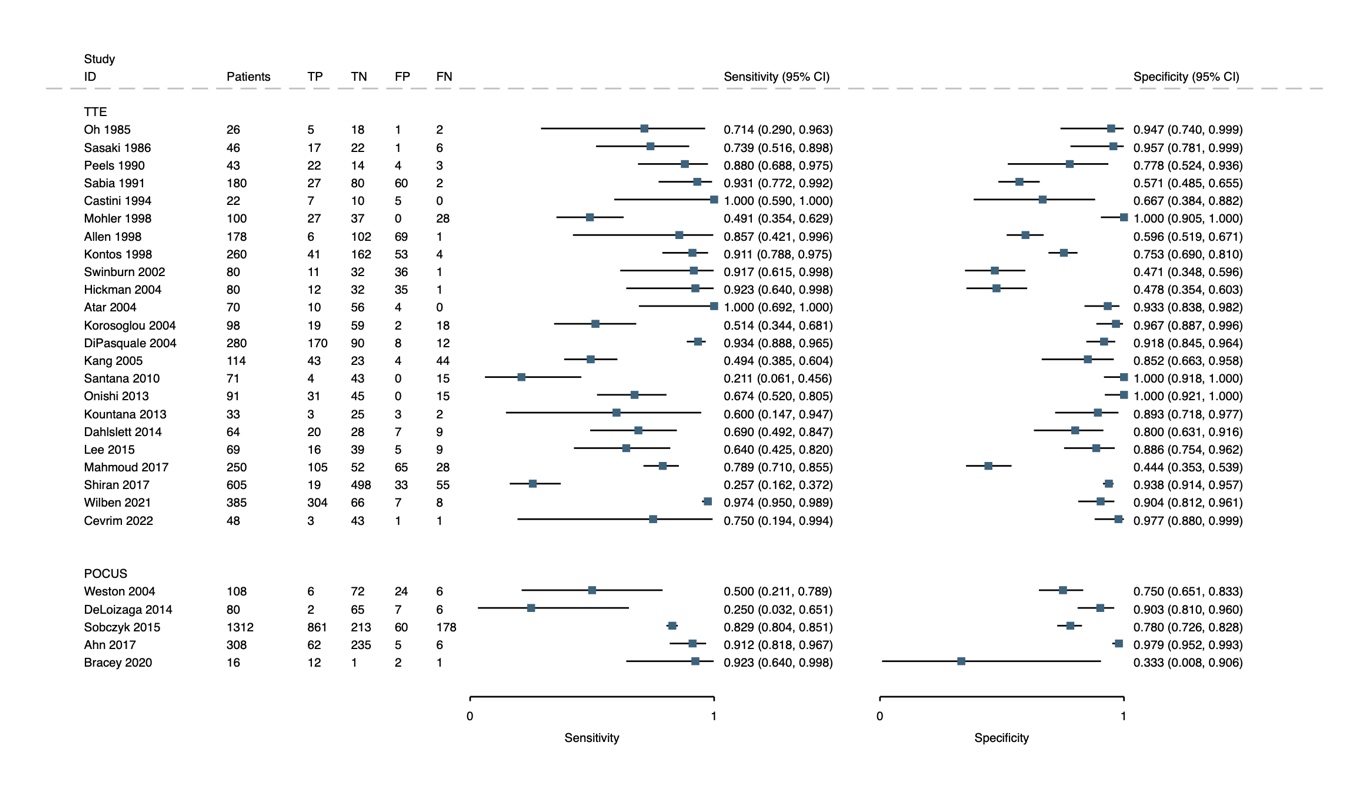


e-Figure 9. Paired Forest plot of sensitivity and specificity by ultrasound scanning protocol. TP= true positive, TN = true negative, FP= false positive, FN= false negative.


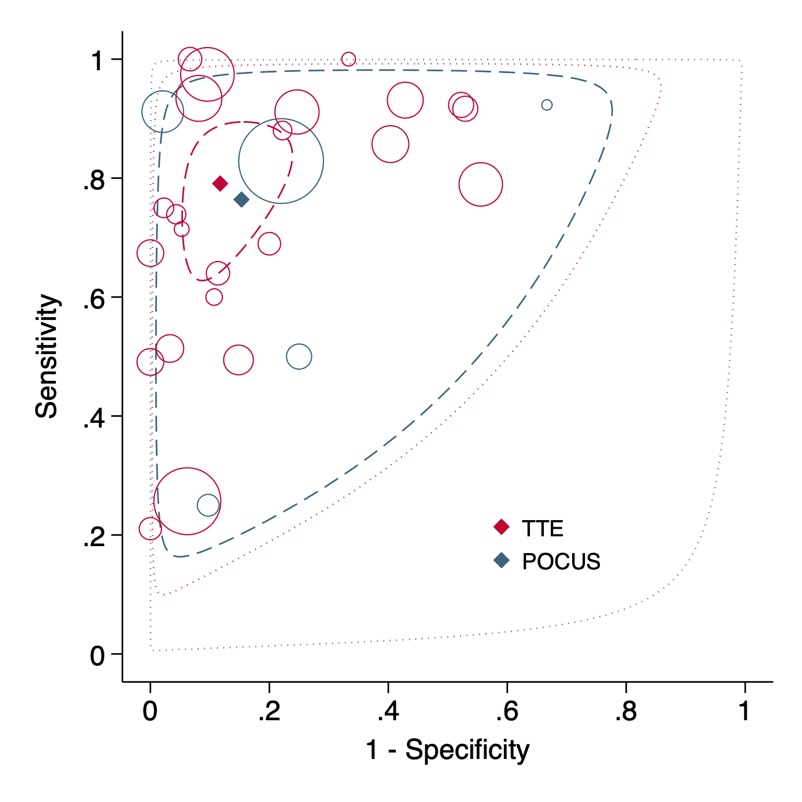


e-Figure 10. SROC plot of sensitivity and specificity by ultrasound scanning protocol. Each study is represented by a circle, with the size of the circle indicating the size of the sample.

## Subgroup analysis by reference standard

Different reference standards were used across studies. Eleven studies used final chart review as the reference standard, seven studies used coronary arteriography, seven studies used combination of different tests and three studies used only cardiac enzymes. Paired Forest plots of sensitivity and specificity for each subgroup are displayed in e-Figure 11. Sensitivity was not significantly different across groups but there was a significant effect of the reference standard on the specificity of cardiac ultrasound. Studies that used final chart review as reference standard, showed a high specificity 93.0% (95%CI 86.7 to 96.4%) with low uncertainty in the estimates, while studies that used cardiac enzymes showed a very low specificity 50.7% (95%CI 23.8 to 77.3%) with very high uncertainty in the estimates, probably reflecting current knowledge that cardiac enzymes may be elevated in myocardial injury of different etiologies not related to ischemia. Studies where the reference standard was coronary angiography had a specificity of 88.7% (95%CI 76.2 to 95%) and studies that used combination of different test showed a specificity of 85.2% (95%CI 70.6 to 93.2), both with moderate degree of uncertainty in the results. There was substantial between-study heterogeneity as depicted in the 95% prediction region across all groups (e-Figure 12).


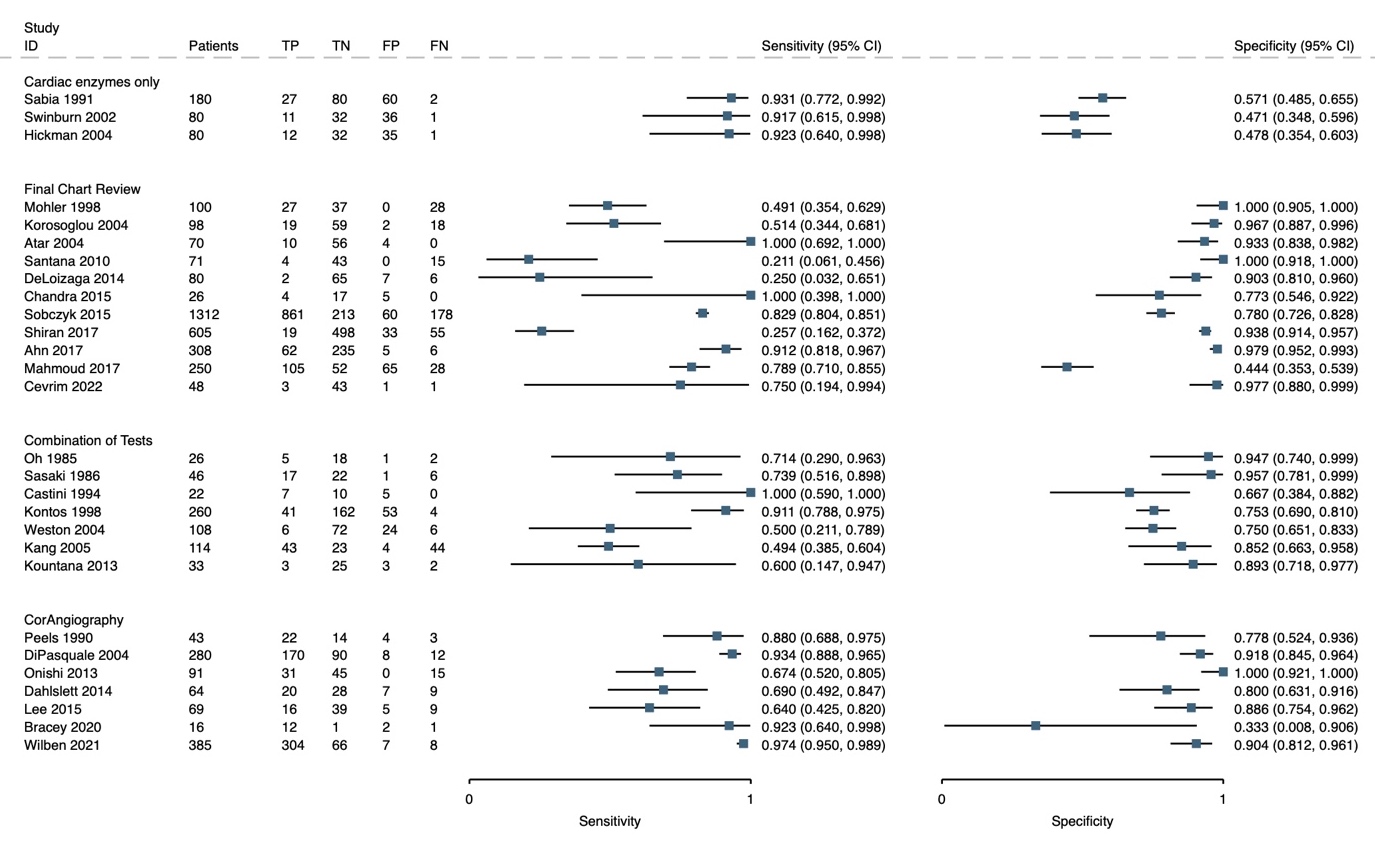


e-Figure 11. Paired Forest plot of sensitivity and specificity by reference standard. TP= true positive, TN = true negative, FP= false positive, FN= false negative.


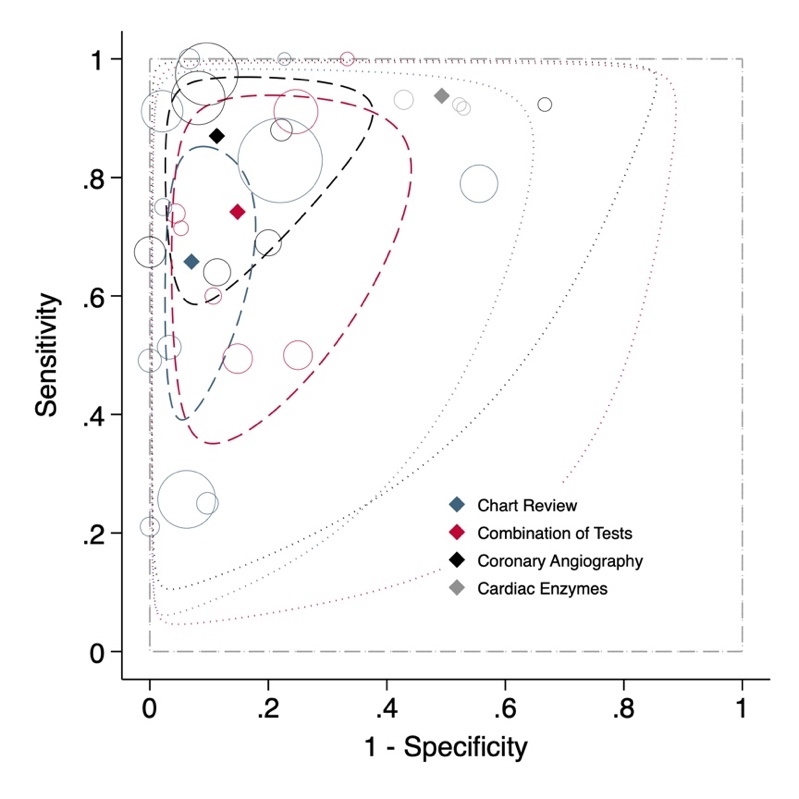


e-Figure 12. SROC plot of sensitivity and specificity by reference standard. Each study is represented by a circle, with the size of the circle indicating the size of the sample.

## Subgroup analysis by target condition

Studies used different target conditions within the spectrum of myocardial ischemia. Eight studies used myocardial infarction, 16 studies used acute coronary syndrome and five studies used significant coronary stenosis. Paired Forest plot is depicted in e-Figure 13. The target condition had a significant effect on sensitivity and specificity across groups. Pooled sensitivity was 90.7% (95%CI 78.3 to 96.3%) in studies that used myocardial infarction as the target condition, 85.0% (95%CI 65.4 to 94.4%) in studies that used significant coronary stenosis and 68.0% (95%CI 53.3 to 79.8%) in studies that used acute coronary syndrome (p-value = 0.035), with a high degree of uncertainty of the estimates in the three groups. Low sensitivity for acute coronary syndrome probably reflects that this syndrome includes patients with both, unstable angina and myocardial infarction, and the transient nature of RWMA in unstable angina that may have already subsided when the ultrasound is performed. Pooled specificity was 93.2% (95%CI 88.2 to 96.2%) for studies that used acute coronary syndrome as the target condition, 82.7% (95%CI 81.5 to 91.2%) for significant coronary stenosis and 67.4% (95%CI 49.4 to 81.4%) for myocardial infarction. Low specificity in the latter group also reflects the pathophysiologic process, where transient myocardial ischemia with transient RMWAs may not result in myocardial necrosis. There is absence of overlap between the 95% confidence regions displayed in the SROC plot (e-Figure 14) for acute coronary syndrome and myocardial infarction, reflecting that significant effect that inclusion or exclusion of unstable angina in the target definition has in the overall diagnostic accuracy. There was substantial between-study heterogeneity in all groups represented in the wide 95% prediction regions.


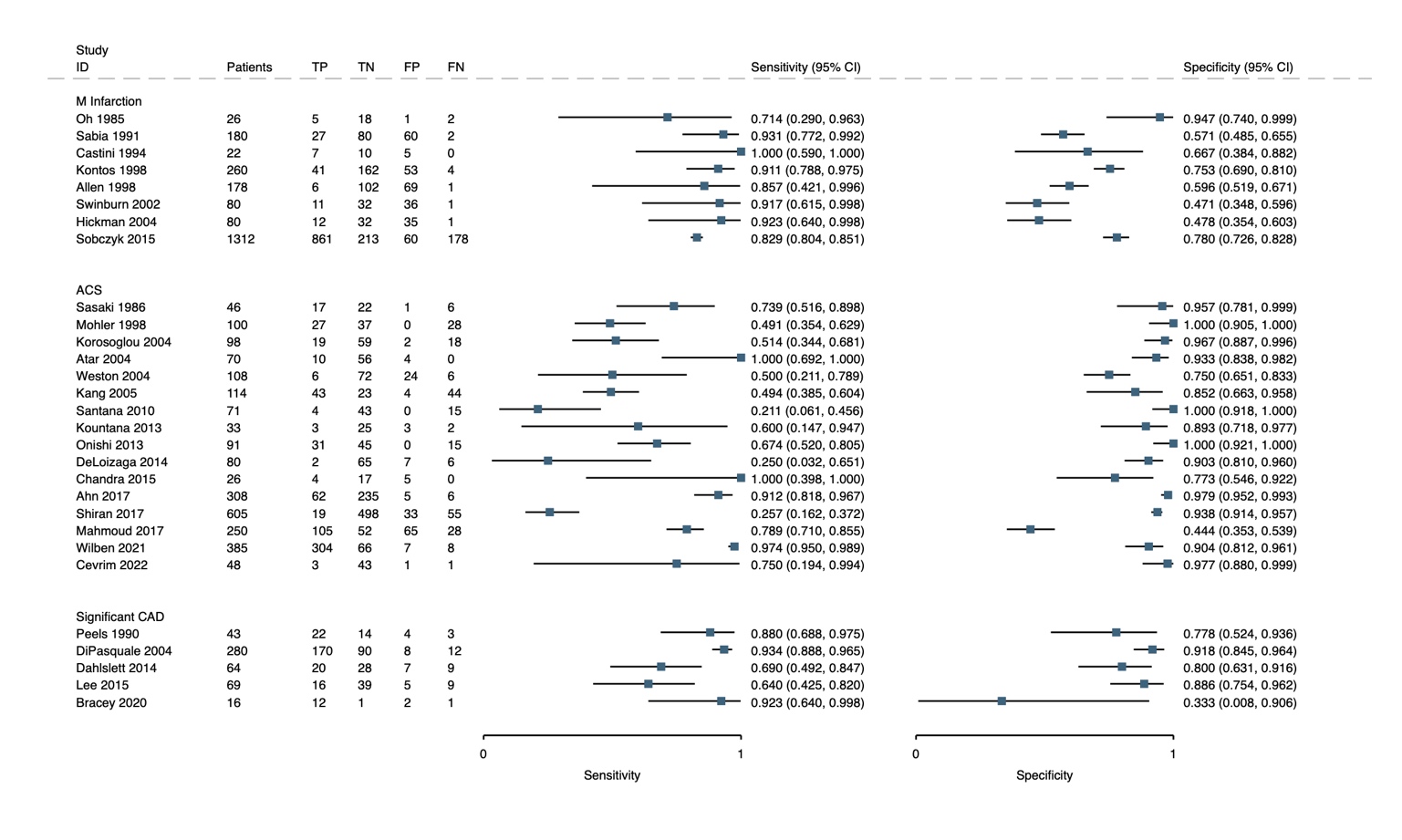


e-Figure 13. Paired Forest plot of sensitivity and specificity by target condition TP= true positive, TN = true negative, FP= false positive, FN= false negative


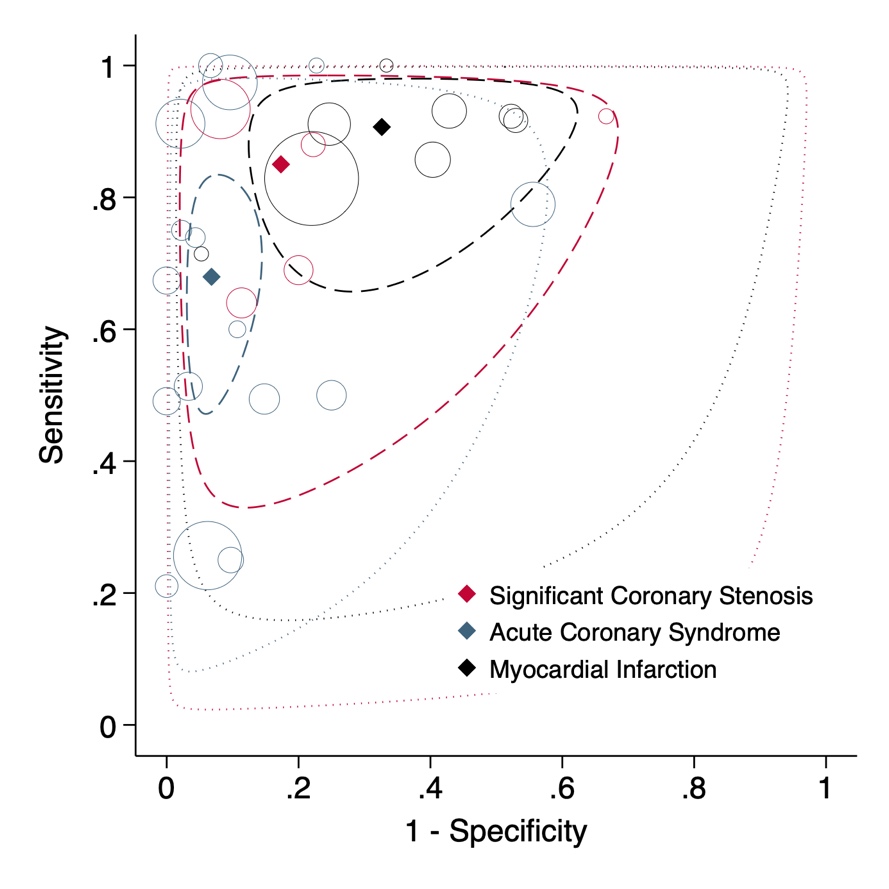


e-Figure 14. SROC plot of sensitivity and specificity by target condition. Each study is represented by a circle, with the size of the circle indicating the size of the sample.

# e-Appendix 8. Sensitivity Analyses

Sensitivity analyses were conducted by removing studies with high risk of bias, studies with inappropriate reference standard and unpublished studies.

## Removing studies with high risk of bias

Risk of bias was assessed by the QUADAS-2 tool, and judged upon four domains: patient selection, index test, reference standard and flow and timing. Sensitivity analysis was conducted by removing 16 studies with high risk of bias in multiple domains (e-Table 3).^3,4,8-10,12,13,15,16,22-28^ Sensitivity was slightly increased, and specificity remained similar (e-Figure 15).

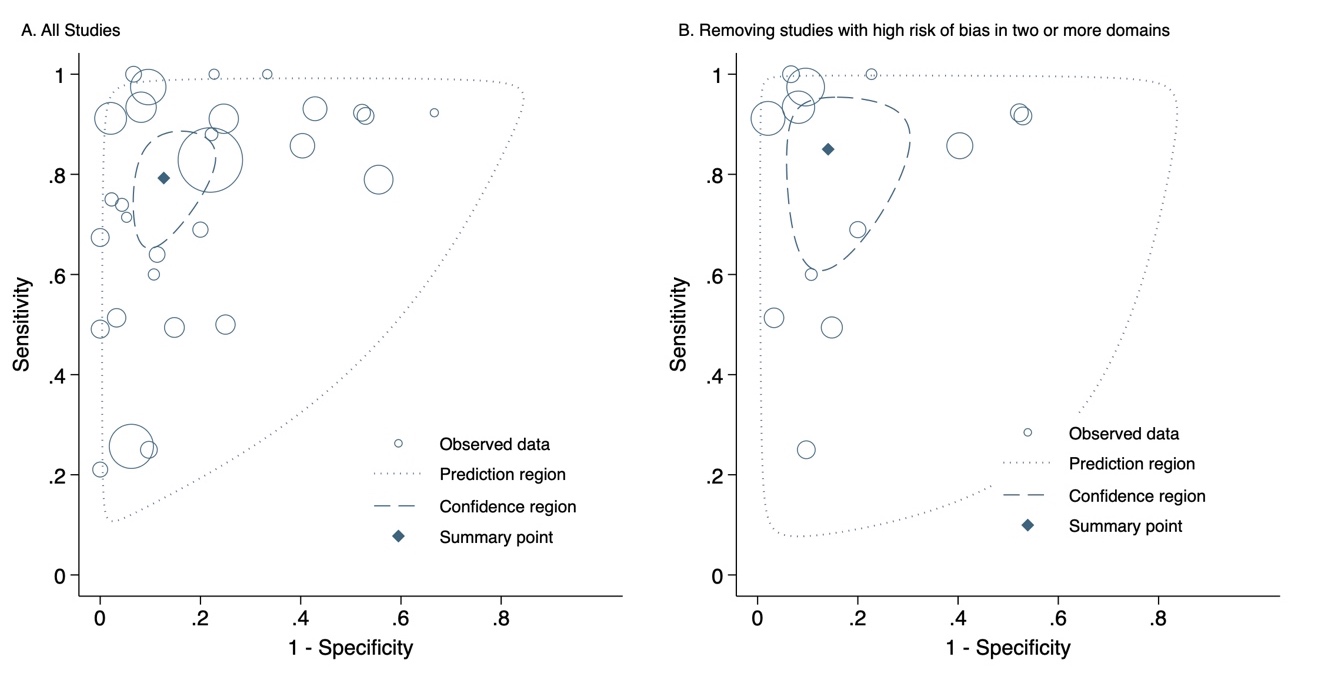


e-Figure 15. Sensitivity analysis by removing studies with high risk of bias in two or more domains. A= SROC with all studies included, B= SROC removing studies an inappropriate reference standard.

## Removing studies with inappropriate reference standard

Current gold standard for myocardial ischemia relies on the fulfillment of a set of criteria which includes cardiac enzymes, clinical presentation, ECG, echocardiographic imaging, and coronary angiogram. Therefore, the reference standard that mostly resembles this gold standard is the final chart review. Coronary angiography for the detection of critical stenosis may also be considered an appropriate gold standard. However, studies that used an arbitrary combination of tests included important variations in the information used in an opinion-based reference standard and may not be considered appropriate. Also, using cardiac enzymes only is not considered appropriate according to current practice. After removing studies with inappropriate reference standards,^3,7,9,13,16,17,20,21,23,27,29^ there was a similar sensitivity and a slightly higher specificity for cardiac ultrasound, with uncertainty of the estimates being slightly higher in sensitivity (e-Table 4, e-Figure 16).

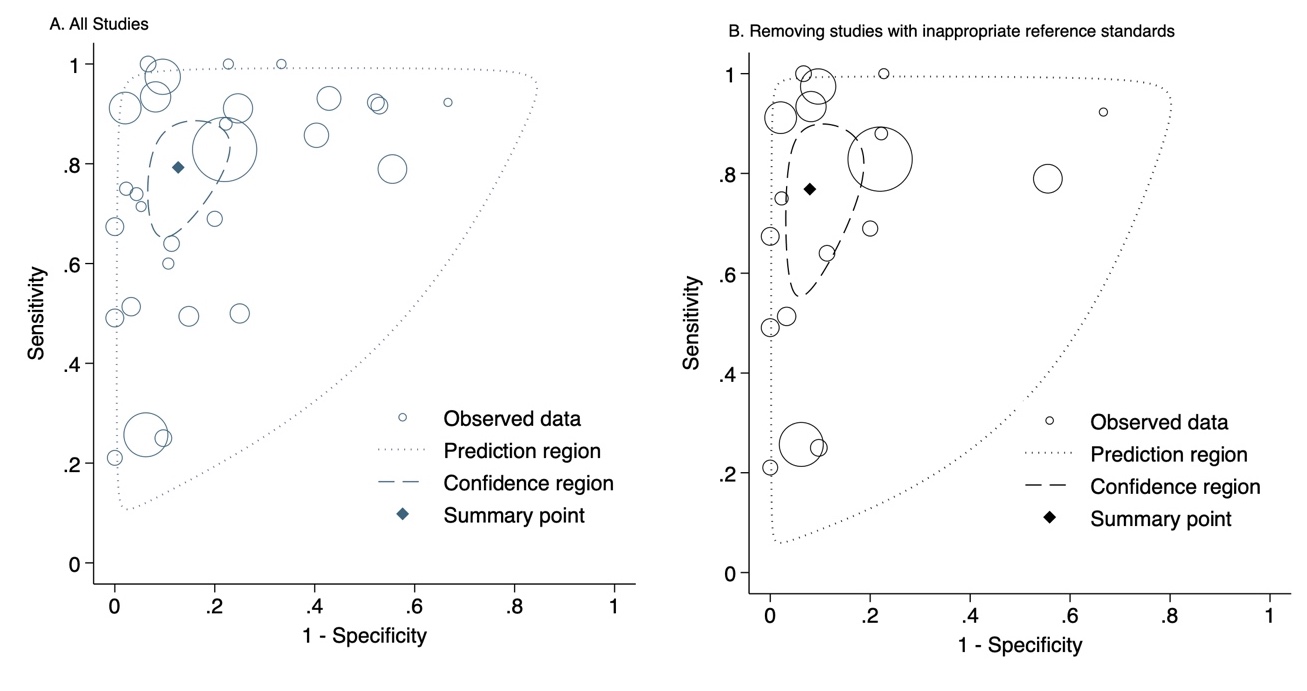


e-Figure 16. Sensitivity analysis by removing studies with an inappropriate reference standard. A= SROC with all studies included, B= SROC removing studies an inappropriate reference standard.

## Removing unpublished studies (conference abstracts).

Some studies were unpublished studies, mainly conference abstracts. These reports lacked a detailed description on important study characteristics. Therefore, effect of this less rigorous studies was assessed by sensitivity analysis (e-Table 5). After removing conference abstracts,^5,12,17,18,24^ sensitivity and specificity did not change, and statistical uncertainty and heterogeneity between studies also remained unchanged as can be observed in the SROC plots (e-Figure 17).

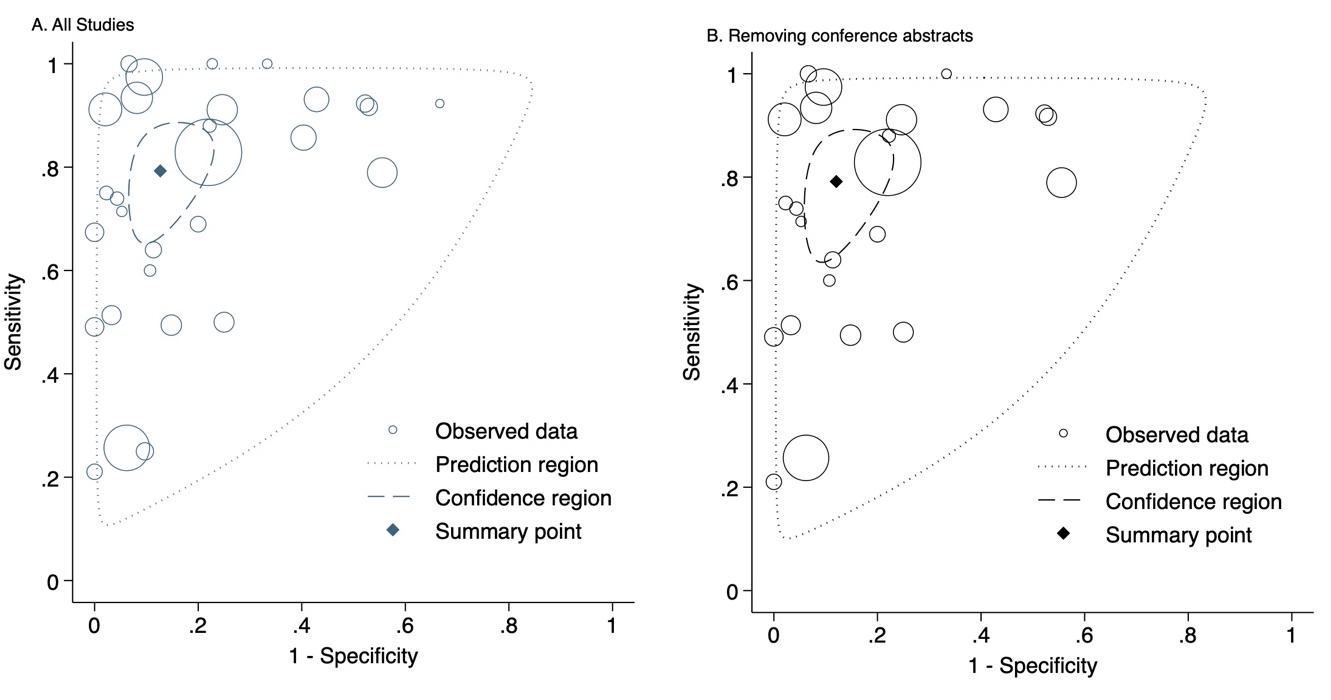


e-Figure 17. Sensitivity analysis by removing studies with an inappropriate reference standard. A= SROC with all studies included, B= SROC removing conference abstracts.

# e-Appendix 9. Publication Bias

Deek’s funnel plot was constructed to assess for publication bias and other sample size related effects, plotting the diagnostic odds ratio and the inverse of the square root of the effective sample size. The funnel plot shows acceptable symmetry with a p value of 0.91 (e-Figure 18), suggesting no publication or small sample size study effect. However, tests of funnel plot asymmetry have reduced power in meta-analyses with high heterogeneity, and therefore results must be interpreted with caution as the exclusion of publication bias may not be possible under these circumstances.^38-40^


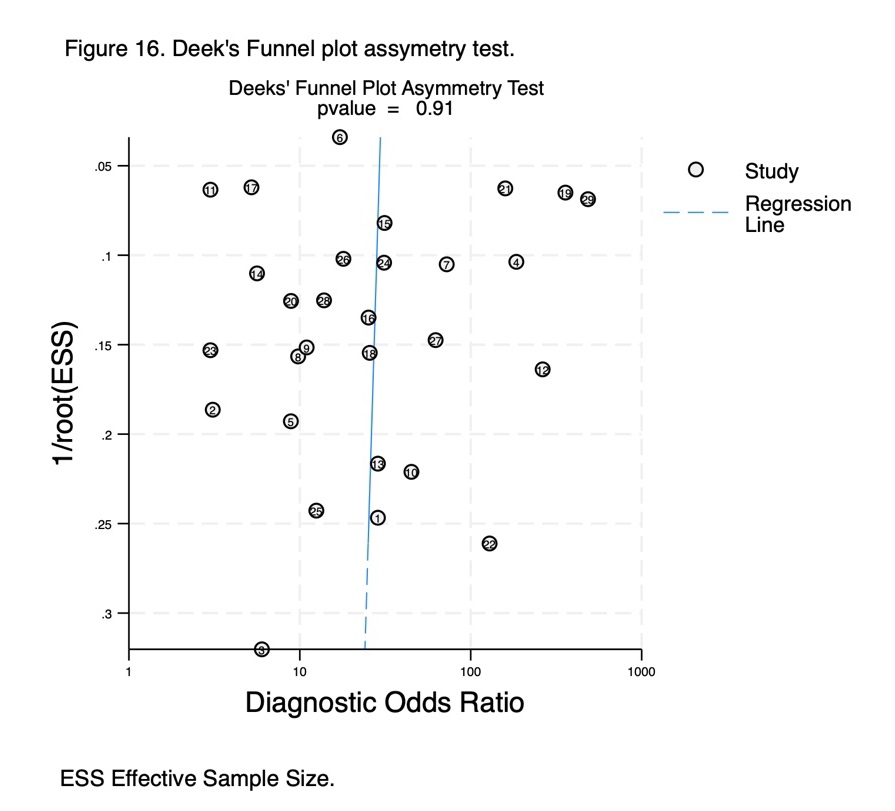


e-Figure 18. Deeks’ Funnel plot of asymmetry test. ESS Effective Sample Size

# e-Appendix 10. Deviations from the original protocol

# REFERENCES

1. Ahn JH, Jeon J, Toh HC, et al. SEARCH 8Es: A novel point of care ultrasound protocol for patients with chest pain, dyspnea or symptomatic hypotension in the emergency department. *PLoS One*. 2017;12(3):e0174581. doi:10.1371/journal.pone.0174581

2. Atar S, Feldman A, Darawshe A, Siegel RJ, Rosenfeld T. Utility and diagnostic accuracy of hand-carried ultrasound for emergency room evaluation of chest pain. *Am J Cardiol*. Aug 1 2004;94(3):408-9. doi:10.1016/j.amjcard.2004.04.052

3. Castini D, Gentile F, Ornaghi M, et al. Utilità dell’ecocardiografia in pronto soccorso per la diagnosi precoce di infarto miocardico acuto. *Il Cuore*. 11(3):243-250.

4. Çevrim Ö, Boydak B, Yürüktümen A, et al. The Diagnostic Value of Echocardiography Performed by an Emergency Medicine Physician in the Diagnosis of Acute Coronary Syndrome: A Comparative Study With Cardiologist. *Journal of Diagnostic Medical Sonography*. 2023;39(2):151-162. doi:10.1177/87564793221138100

5. De Loizaga SL, T.; Dagan, A.; Pacheco, F.; Thomas, D.; Oligino, E.; Herbst, M. Focused echocardiograms by emergency physicians for the risk stratification of patients with chest pain. *Academic Emergency Medicine*. May 2014;1:s36.

6. Di Pasquale P, Cannizzaro S, Scalzo S, et al. Sensitivity, specificity and predictive value of the echocardiography and troponin-T test combination in patients with non-ST elevation acute coronary syndromes. *Int J Cardiovasc Imaging*. Feb 2004;20(1):37-46.

7. Kang DH, Kang SJ, Song JM, et al. Efficacy of myocardial contrast echocardiography in the diagnosis and risk stratification of acute coronary syndrome. *Am J Cardiol*. Dec 1 2005;96(11):1498-502. doi:10.1016/j.amjcard.2005.07.057

8. Mahmoud MZ. Echocardiography in the Evaluation of Chest Pain in the Emergency Department. *Pol J Radiol*. 2017;82:798-805. doi:10.12659/PJR.904031

9. Sabia P, Afrookteh A, Touchstone DA, Keller MW, Esquivel L, Kaul S. Value of regional wall motion abnormality in the emergency room diagnosis of acute myocardial infarction. A prospective study using two-dimensional echocardiography. *Circulation*. Sep 1991;84(3 Suppl):I85-92.

10. Sobczyk D, Nycz K, Andruszkiewicz P. Validity of a 5-minute focused echocardiography with A-F mnemonic performed by non-echocardiographers in the management of patients with acute chest pain. *Cardiovasc Ultrasound*. Mar 26 2015;13:16. doi:10.1186/s12947-015-0010-y

11. Wilben V, Limbad D, Bs B, Ts S, Kanchi M. Recommendation for Inclusion of Surface Echocardiography in Evaluation of Chest Pain in Acute Emergency Care. *Journal of Cardiac Critical Care TSS*. 2021/08/18 2021;5(02):097-102. doi:10.1055/s-0041-1723930

12. Bracey AM, M.; Massey, L.; Singer, A. J.; Alian, A.; Secko, M. Point-of-care ultrasound detects regional wall motion abnormalities and acute coronary occlusions. *Academic Emergency Medicine*. 2020;27(S1):86.

13. Kontos MC, Arrowood JA, Jesse RL, et al. Comparison between 2-dimensional echocardiography and myocardial perfusion imaging in the emergency department in patients with possible myocardial ischemia. *Am Heart J*. Oct 1998;136(4 Pt 1):724-33. doi:10.1016/s0002-8703(98)70022-5

14. Korosoglou G, Labadze N, Hansen A, et al. Usefulness of real-time myocardial perfusion imaging in the evaluation of patients with first time chest pain. *Am J Cardiol*. Nov 15 2004;94(10):1225-31. doi:10.1016/j.amjcard.2004.07.104

15. Mohler ER, 3rd, Ryan T, Segar DS, et al. Clinical utility of troponin T levels and echocardiography in the emergency department. *Am Heart J*. Feb 1998;135(2 Pt 1):253-60. doi:10.1016/s0002-8703(98)70090-0

16. Weston P, Alexander JH, Patel MR, Maynard C, Crawford L, Wagner GS. Hand-held echocardiographic examination of patients with symptoms of acute coronary syndromes in the emergency department: the 30-day outcome associated with normal left ventricular wall motion. *Am Heart J*. Dec 2004;148(6):1096-101. doi:10.1016/j.ahj.2004.05.026

17. Allen MO, J. K.; Farkouh, M. E.; Zinsmeister, A. R.; Smars, P. A.; Seward, J. B.; Reeder, G. S. The rate of emergency room echocardiography in imaging patients with chest pain at intermediate risk for acute myocardial infarction: A substudy of CHEER. presented at: Journal of the American College of Cardiology; 1998;

18. Chandra AB, U.; Kurkowski, E.; Menlove, S.; Vermeulen, M.; Carmody, K. 356 Emergency Physician-Performed Echocardiography as a Predictor of Cardiac Events in Patients Presenting With Symptoms of Acute Coronary Syndrome. *Annals of Emergency Medicine*. 2015;66(S):128-129.

19. Dahlslett T, Karlsen S, Grenne B, et al. Early assessment of strain echocardiography can accurately exclude significant coronary artery stenosis in suspected non-ST-segment elevation acute coronary syndrome. *J Am Soc Echocardiogr*. May 2014;27(5):512-9. doi:10.1016/j.echo.2014.01.019

20. Hickman M, Swinburn JM, Senior R. Wall thickening assessment with tissue harmonic echocardiography results in improved risk stratification for patients with non-ST-segment elevation acute chest pain. *Eur J Echocardiogr*. Mar 2004;5(2):142-8. doi:10.1016/S1525-2167(03)00077-5

21. Kountana E, Tziomalos K, Semertzidis P, et al. Comparison of the diagnostic accuracy of ischemia-modified albumin and echocardiography in patients with acute chest pain. *Exp Clin Cardiol*. Spring 2013;18(2):98-100.

22. Lee M, Chang SA, Cho EJ, et al. Role of strain values using automated function imaging on transthoracic echocardiography for the assessment of acute chest pain in emergency department. *Int J Cardiovasc Imaging*. Mar 2015;31(3):547-56. doi:10.1007/s10554-015-0588-z

23. Oh JKS, C.; Miller Jr, F. A. Role of two-dimensional echocardiography in the emergency room. *Echocardiography*. 1985;3:217-226.

24. Onishi TW, T.; Fujita, M.; Mizukami, Y.; Sakata, Y.; Nakatani, S.; Nanto, S.; Uematsu, M. Risk stratification of chest pain in emergency department using non-provocative echocardiography combined with tissue doppler dyssynchrony imaging. *European heart journal cardiovascular Imaging*. 2013;2:ii106.

25. Peels CH, Visser CA, Kupper AJ, Visser FC, Roos JP. Usefulness of two-dimensional echocardiography for immediate detection of myocardial ischemia in the emergency room. *Am J Cardiol*. Mar 15 1990;65(11):687-91. doi:10.1016/0002-9149(90)90143-o

26. Santana G, Castro M, Luiz D, Gomes M, Andrade N, Natividade J. Papel do Ecocardiograma Transtorácico de Rotina na Unidade de Dor Torácica. *Rev bras ecocardiogr imagem cardiovasc*. 2010;23(1):26-30.

27. Sasaki H, Charuzi Y, Beeder C, Sugiki Y, Lew AS. Utility of echocardiography for the early assessment of patients with nondiagnostic chest pain. *Am Heart J*. Sep 1986;112(3):494-7. doi:10.1016/0002-8703(86)90512-0

28. Shiran A, Blondheim DS, Shimoni S, et al. Two-dimensional strain echocardiography for diagnosing chest pain in the emergency room: a multicentre prospective study by the Israeli echo research group. *Eur Heart J Cardiovasc Imaging*. Sep 1 2017;18(9):1016-1024. doi:10.1093/ehjci/jew168

29. Swinburn JM, Stubbs P, Soman P, Collinson P, Lahiri A, Senior R. Independent value of tissue harmonic echocardiography for risk stratification in patients with non-ST-segment elevation acute chest pain. *J Am Soc Echocardiogr*. Oct 2002;15(10 Pt 1):1031-7. doi:10.1067/mje.2002.121809

30. Rutjes AW, Reitsma JB, Di Nisio M, Smidt N, van Rijn JC, Bossuyt PM. Evidence of bias and variation in diagnostic accuracy studies. *CMAJ*. Feb 14 2006;174(4):469-76. doi:10.1503/cmaj.050090

31. Hall MK, Kea B, Wang R. Recognising Bias in Studies of Diagnostic Tests Part 1: Patient Selection. *Emerg Med J*. Jul 2019;36(7):431-434. doi:10.1136/emermed-2019-208446

32. Whiting PF, Rutjes AW, Westwood ME, et al. QUADAS-2: a revised tool for the quality assessment of diagnostic accuracy studies. *Ann Intern Med*. Oct 18 2011;155(8):529-36. doi:10.7326/0003-4819-155-8-201110180-00009

33. Schunemann HJ, Mustafa RA, Brozek J, et al. GRADE guidelines: 21 part 2. Test accuracy: inconsistency, imprecision, publication bias, and other domains for rating the certainty of evidence and presenting it in evidence profiles and summary of findings tables. *J Clin Epidemiol*. Jun 2020;122:142-152. doi:10.1016/j.jclinepi.2019.12.021

34. Schunemann HJ, Mustafa RA, Brozek J, et al. GRADE guidelines: 21 part 1. Study design, risk of bias, and indirectness in rating the certainty across a body of evidence for test accuracy. *J Clin Epidemiol*. Jun 2020;122:129-141. doi:10.1016/j.jclinepi.2019.12.020

35. *GRADEpro GDT: GRADEpro Guideline Development Tool [Software]*. McMaster University and Evidence Prime; 2022. Available from gradepro.org

36. Hauser AM, Gangadharan V, Ramos RG, Gordon S, Timmis GC. Sequence of mechanical, electrocardiographic and clinical effects of repeated coronary artery occlusion in human beings: echocardiographic observations during coronary angioplasty. *J Am Coll Cardiol*. Feb 1985;5(2 Pt 1):193-7.

37. Nesto RW, Kowalchuk GJ. The ischemic cascade: temporal sequence of hemodynamic, electrocardiographic and symptomatic expressions of ischemia. *Am J Cardiol*. Mar 9 1987;59(7):23C-30C. doi:10.1016/0002-9149(87)90192-5

38. van Enst WA, Ochodo E, Scholten RJ, Hooft L, Leeflang MM. Investigation of publication bias in meta-analyses of diagnostic test accuracy: a meta-epidemiological study. *BMC Med Res Methodol*. May 23 2014;14:70. doi:10.1186/1471-2288-14-70

39. Macaskill P TY, Deeks JJ, Gatsonis C. Chapter 9: Understanding meta-analysis. Draft version (4 October 2022) for inclusion. In: Deeks JJ BP, Leeflang MM, Takwoingi Y, editor(s). ed. *Cochrane Handbook for Systematic Reviews of Diagnostic Test Accuracy Version 2*. London: Cochrane.

40. Deeks JJ, Macaskill P, Irwig L. The performance of tests of publication bias and other sample size effects in systematic reviews of diagnostic test accuracy was assessed. *J Clin Epidemiol*. Sep 2005;58(9):882-93. doi:10.1016/j.jclinepi.2005.01.016
